# Exploring LCST- and UCST-like Behavior of Branched Molecules Bearing Repeat Units of Elastin-like Peptides as Side Components

**DOI:** 10.1021/acs.biomac.4c00751

**Published:** 2024-10-09

**Authors:** Naoki Tanaka, Keitaro Suyama, Keisuke Tomohara, Takeru Nose

**Affiliations:** † Department of Chemistry, Faculty and Graduate School of Science, Kyushu University, Fukuoka 819-0395, Japan; ‡ Faculty of Arts and Science, Kyushu University, Fukuoka 819-0395, Japan; § Faculty and Graduate School of Pharmaceutical Sciences, Kyoto Pharmaceutical University, Kyoto 607-8412, Japan

## Abstract

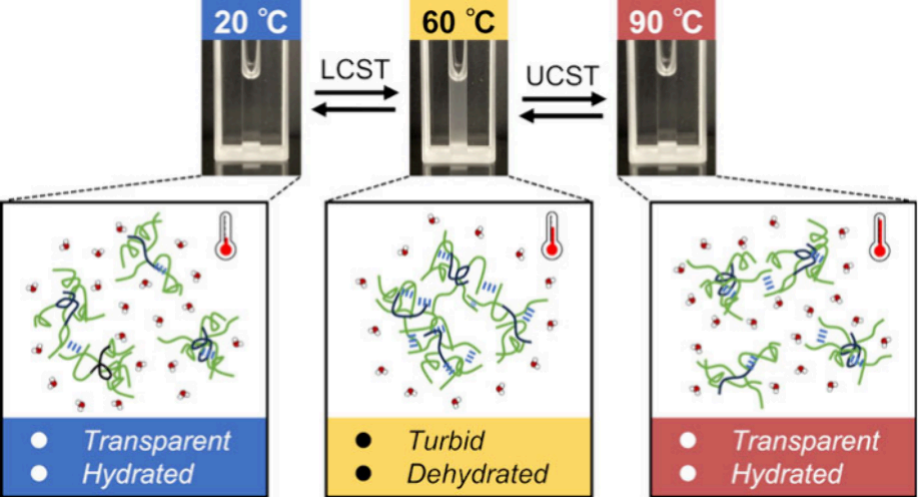

Elastin-like peptides (ELPs) exhibit lower critical solution temperature (LCST)-type behavior, being soluble at low temperatures and insoluble at high temperatures. While the properties of linear, long-chain ELPs are well-studied, short-chain ELPs, especially those with branched architectures, have been less explored. Herein, to obtain further insights into multimeric short ELPs, we investigated the temperature-responsive properties of branched molecules composed of a repeating pentapeptide unit of short ELPs, Phe-Pro-Gly-Val-Gly, as side components and oligo(Glu) as a backbone structure. In turbidimetry experiments, the branched ELPs showed LCST-like behavior similar to conventional ELPs and upper critical solution temperature (UCST)-like behavior, which are rarely observed in ELPs. In addition, the morphological aspects and mechanisms underlying the temperature-responsiveness were investigated. We observed that spherical aggregates formed, and the branched ELPs underwent structural changes through the self-assembly process. This study demonstrates the unique temperature-responsiveness of branched short ELPs, providing new insights into the future development and use of ELPs with tailored properties.

## Introduction

Stimuli-responsive materials dynamically alter their physical or chemical properties in response to external or internal stimuli such as temperature changes, pH variations, light exposure, ionic strength, or mechanical force.^1,2^ Among them, temperature-responsive molecules have garnered extensive attention because of the ease of controlling the temperature to trigger the alteration of properties in an on-demand and remote manner.^1,3,4^ Temperature-responsive behavior is typically classified into two types: lower critical solution temperature (LCST) and upper critical solution temperature (UCST).^5^ LCST polymers are soluble at low temperatures and insoluble at high temperatures, whereas UCST polymers are insoluble at low temperatures and soluble at high temperatures.

Elastin-like peptides (ELPs) are synthetic peptides that mimic the primary sequence of the hydrophobic domain of tropoelastin. They exhibit LCST-like behavior (also known as coacervation) in aqueous solutions, and their stimuli-responsive behavior, in combination with their potential biodegradability and biocompatibility,^6,7^ has made them particularly attractive as responsive biomaterials for protein functionalization, purification supports, drug carriers, and scavengers of toxic substances.^8−11^ ELPs are typically composed of a sequence commonly identified in vertebrates,^12−14^ (Val-Pro-Gly-Xaa-Gly)_*n*_, where Xaa is any amino acid except Pro. The LCST-like behavior of ELPs can be modified by varying parameters such as the amino acid composition, peptide chain length (repetition number), peptide concentration, salt present in the solution, salt concentration, pH, and solvent composition.^15−23^

Among the different approaches to tuning the temperature-responsiveness of ELPs, short-chain ELPs offer a unique advantage in that during the chemical synthesis process, they allow for the replacement, addition, or deletion of a wide variety of amino acids (including d-amino acids or unnatural amino acids) at any position in their sequence.^24^ This customizability enables precise control over their coacervation behavior for specific needs. Previously, we developed an ELP composed of (Phe-Pro-Gly-Val-Gly)_*n*_ (F*n*, where *n* stands for repetition number) and found that F*n* exhibits LCST-like behavior with *n* = approximately 5.^25^ Following this finding, we developed branched ELPs ranging from dimeric to tetrameric with *n* = 1–5 using chemical synthetic approaches, demonstrating that multimeric F*n* molecules exhibit higher coacervation tendencies than their constituent monomeric F*n* molecules.^24,26,27^ However, the properties of branched short ELPs, especially those with *n* = 1, are relatively less investigated than those of linear polypentapeptide ELPs.

In our recent study, we showed that branched ELPs comprising four F1 chains exhibited LCST-like behavior, although the monomeric pentapeptide itself did not show coacervation because of its shortness.^24^ In the study, however, we found that structural changes induced by temperature changes in the temperature-responsive branched multimer and linear F4 were similar to those of the monomer pentapeptide: they show a polyproline type II (PPII) helix at low temperatures, whereas they have a higher preference for β-turn or β-sheet structures at high temperatures. Short ELPs with a (VPGVG)_*n*_ sequence have been reported to show the same structural changes upon temperature change as long linear (VPGVG)_*n*_,^28^ and there have been several studies investigating brush-shaped branched polymers with VPGVG chains.^29−33^ Although ELPs of different sequences can show different structural features, the structural similarity found between a pentapeptide unit and its polymer suggests that branched polymers bearing the pentapeptide chains as side groups would show coacervation similar to that of linear ELPs. Accordingly, we hypothesized that branched molecules comprising F1 chains can readily exhibit temperature-responsive behavior.

Herein, based on this hypothesis, we synthesized a series of branched peptides consisting of oligo(Glu) as the backbone structure and F1 chains as side components to expand our understanding of branched short ELPs. The obtained branched short ELPs were subjected to turbidity measurements to investigate their temperature-responsiveness, and we found that they exhibited LCST-like behavior depending on the temperature change at a peptide concentration of 0.5–4 mM. Additionally, at 0.5 mM, the branched ELPs with six F1 chains showed UCST-like behavior, which is rarely observed in ELPs. The mechanism behind the temperature-responsiveness was investigated using circular dichroism (CD) measurement, the thioflavin T (ThT) assay, and molecular dynamics (MD) simulation.

## Materials and Methods

### Chemicals

9-Fluorenylmethyloxycarbonyl (Fmoc)-Phe-OH, Fmoc-Pro-OH, Fmoc-Gly-OH, Fmoc-Val-OH, Fmoc-NH-SAL-MBHA resin (100–200 mesh), *N*,*N*-diisopropylethylamine (DIPEA), piperidine, trifluoroacetic acid (TFA), 1-[(1-(cyano-2-ethoxy-2-oxoethylideneaminooxy)dimethylaminomorpholino)]uronium hexafluorophosphate (COMU), 2-(1*H*-benzotriazole-1-yl)-1,1,3,3-tetramethyluronium hexafluorophosphate (HBTU), and OxymaPure were purchased from Watanabe Chemical Industries (Hiroshima, Japan). Fmoc-Glu(O^*t*^Bu)-OH, Fmoc-Glu-O^*t*^Bu, and triisopropylsilane (TIS) were purchased from Tokyo Chemical Industry (Tokyo, Japan). *N*,*N*-Dimethylformamide (DMF) was purchased from Kanto Chemical (Tokyo, Japan). Water for the experiment was purified using a Milli-Q Integral 3 instrument (Merck Millipore, Darmstadt, Germany). Other solvents and reagents were obtained from commercial suppliers and used without further purification.

### Peptide Synthesis and Purification

The linear peptides were obtained as follows. H-FPGVG-NH_2_ (F1) was obtained by solid-phase synthesis based on the Fmoc strategy with HBTU/OxymaPure on CSBio II, an automated peptide synthesizer (Menlo Park, CA). After the cleavage with a solution of 95% TFA, 2.5% TIS, and 2.5% H_2_O, the TFA solution was purified with a Sep-Pak cartridge (C18, 10 g), followed by lyophilization to give F1 as a colorless solid. Backbone oligopeptides, Fmoc-[α-(E(OH)]_*n*_-FPGVG-NH_2_ and Fmoc-[γ-(E(OH)]_*n*_-FPGVG-NH_2_ (*n* = 4, 5, or 6), were also obtained by solid-phase synthesis on CSBio II. After cleavage with a solution of 95% TFA, 2.5% TIS, and 2.5% H_2_O, cold diethyl ether was added to the solution. Then the precipitate was dried under vacuum to give the oligopeptide as a colorless solid.

The branched ELPs were obtained as follows. Fmoc-[E(OH)]_*n*_-FPGVG-NH_2_ (0.02 mmol), F1 (*n* × 0.03 mmol, i.e., 1.5 equiv per carboxyl group), COMU (*n* × 0.04 mmol), and DIPEA (*n* × 0.04 mmol) were dissolved in 200 μL of DMF, and the solution was kept stirred at room temperature for a day. Then, additional COMU and DIPEA (*n* × 0.04 mmol each) were added, and the solution was kept stirred for 2 days. Then, 200 μL of water was added, and the solution was stirred for 1 h. To the solution was added piperidine to give 20% (v/v) piperidine solution and kept stirred overnight. The reaction mixture was filtered and applied to Sep-Pak (C8, 2 g) for prepurification and purified with RP-HPLC (JASCO PU-2089 equipped with UV-2075 or JASCO PU-4180 equipped with UV-4075, JASCO, Tokyo, Japan) using C8 columns (COSMOSIL 5C8-AR-300 Packed Column, 20 mm I.D. × 150 mm, Nacalai Tesque Inc., Kyoto, Japan). The solvent system for RP-HPLC consisted of 0.1% TFA aqueous solution (v/v, solvent A) and a mixture of 80% acetonitrile and 20% solvent A (v/v, solvent B). Then the purified fractions were evaporated and lyophilized to give the target branched ELP, [α- or γ-E(F1)]_*n*_-F1 (H-[E(FPGVG-NH_2_)]_*n*_-FPGVG-NH_2_), as a colorless solid.

The peptides obtained by the above methods were analyzed for their purity by an ACQUITY UPLC H-Class (Waters Co., Milford, MA) equipped with an ACQUITY UPLC BEH C-18 column (100 mm, Waters Co.). The solvent system for UPLC consisted of a 0.1% formic acid aqueous solution (v/v) and 0.1% formic acid in acetonitrile (v/v).

### Turbidity Measurement

Temperature-responsive properties (LCST/UCST-like behavior and its reversibility) were investigated by using a JASCO V-660 spectral photometer (JASCO, Tokyo, Japan). Each peptide was dissolved in phosphate buffer containing NaCl (pH 7.4, 27.4 mM Na_2_HPO_4_, 17.8 mM NaH_2_PO_4_, 1 M NaCl). NaCl in this concentration is used as an additive to trigger an LCST-like behavior for the inverse transition cycling (a method to purify a protein exploiting the LCST-like behavior of ELPs).^9^ The turbidity at 400 nm of each branched ELP solution was traced while increasing or decreasing the temperature at a rate of 0.5 °C/min, except for samples at 0.5 mM, which were traced at 1 °C/min for heating–cooling cycle measurements. For the samples at 0.5 mM, after heating to 90 °C or cooling to 5 °C, the samples were vortexed to eliminate any possible concentration gradients caused by peptide precipitation and kept at 90 or 5 °C for 30 min before starting the subsequent cooling or heating, respectively. The transition temperature (*T*_t_) for LCST was defined as a temperature at which the turbidity of the solution reaches half the maximum value while increasing the temperature. Measurements were performed at least three times.

### DLS Measurement

The particle size distribution of branched ELPs was analyzed in filtered phosphate buffer (pH 7.4, 27.4 mM Na_2_HPO_4_, 17.8 mM NaH_2_PO_4_) containing 1 M NaCl using DLS with a Zetasizer Nano ZS (Malvern Instruments, Worcestershire, U.K.) DLS analysis was performed at temperatures ranging from 15 to 55 °C with 10 °C intervals for [α-E(F1)]_*n*_-F1 and [γ-E(F1)]_*n*_-F1 under conditions where the ELPs have *T*_t_ values around 30 °C for LCST-like behavior (*n* = 4, 2.5 mM; *n* = 5, 1 mM; *n* = 6, 0.5 mM). Additionally, a solution of [α-E(F1)]_5_-F1 at 0.5 mM was analyzed at 20, 60, and 90 °C. The measurement duration was selected automatically. The parameter data set “protein” (data set: refractive index, 1.450; absorption, 0.001) was used as the material parameter, and the parameter data set “water” (data set: refractive index, 1.330; viscosity, 0.8872) was chosen as the dispersant parameter. Attenuation was selected automatically. The autocorrelation curves are shown in Figures S11 and S14.

### Bright-Field Microscopy

The ELP aggregates were observed by using a Leica DM IL LED microscope (Leica Microsystems CMS GmbH, Wetzlar, Germany) equipped with a HI PLAN 40× oil objective (Leica Microsystems CMS GmbH) and an HC PLAN 10× eyepiece (Leica Microsystems CMS GmbH). [α-E(F1)]_*n*_-F1 and [γ-E(F1)]_*n*_-F1 were dissolved in phosphate buffer (pH 7.4, 27.4 mM Na_2_HPO_4_, 17.8 mM NaH_2_PO_4_) containing 1 M NaCl under conditions where the ELPs have *T*_t_ values around 30 °C for LCST-like behavior. Sample imaging was performed at 15 and 45 °C using a Thermo Plate TP-CHSQM (Tokai Hit, Shizuoka, Japan). Additionally, a solution of [α-E(F1)]_5_-F1 at 0.5 mM was prepared in the phosphate buffer containing 1 M NaCl. Sample imaging was performed at 60 °C heated from 15 °C (LCST-like behavior) or cooled from 90 °C (UCST-like behavior) using a Thermo Plate TP-CHSQM (Tokai Hit). Sample imaging was performed after 2 min of equilibration and conducted over 5 min with images taken every minute. The objects at the bottom surface of the slide were observed: these objects were either formed on the glass surface or precipitated there. White balance was adjusted on a Leica Application Suite X (Leica Microsystems CMS GmbH).

### CD Measurement

CD measurements were carried out using a J-725 spectropolarimeter (JASCO) for [α-E(F1)]_5_-F1 and [γ-E(F1)]_5_-F1 in a cuvette with a path length of 1.0 mm. Each peptide was dissolved in filtered phosphate buffer (pH 7.4, 27.4 mM Na_2_HPO_4_, and 17.8 mM NaH_2_PO_4_) at a concentration of 0.1 mg/mL. At this peptide concentration and ionic strength, apparent aggregation behavior was not observed. Spectra were obtained from 190 to 260 nm at temperatures ranging from 10 to 90 °C with 20 °C intervals. Each measurement was performed after 3 min of equilibration at the target temperature. After subtracting the background spectra, Savitzky–Golay filters were applied to smooth all spectra.

### ThT Assay

A ThT fluorescence assay was conducted using an FP-8500 fluorescence spectrometer (JASCO Co.). The phosphate buffer solutions (pH 7.4, 27.4 mM Na_2_HPO_4_, 17.8 mM NaH_2_PO_4_) of [α-E(F1)]_5_-F1 (1 or 0.5 mM) containing 1 M NaCl and 50 μM ThT were prepared. The temperature change rates were the same as those in the turbidity measurements. Using an excitation wavelength of 446 nm, the ThT fluorescence intensities at 483 nm upon temperature changes or over time were recorded.

### MD Simulation

MD simulations of a single molecule were performed for [α-E(F1)]_5_-F1, [γ-E(F1)]_5_-F1, and Ac-F1-NH_2_ using a DELL PRECISION T3610 workstation (Dell Inc., Round Rock, TX). The initial conformations of peptides were generated by Discovery studio 4.0 software (Dassault Systemes BIOVIA, San Diego, CA). The MD simulations were performed in GROMACS 2019 with the AMBER99SB-ILDN force field and the TIP3P explicit solvent model.^34^ The trajectories of these peptides were obtained at simulation temperatures of 278, 303, 333, and 363 K (5, 30, 60, and 90 °C) for 30 ns for the branched ELPs and for 100 ns for Ac-F1-NH_2_. Then, the dihedral angles, radius of gyration (*R*_g_), solvent accessible surface areas (SASA), the number of intramolecular hydrogen bonds (*n*_PP_), and the number of hydrogen bonds between water and peptide (*n*_PW_) were analyzed with omission of the first 10 ns. The detailed calculation protocols are described in the Supporting Information.

## Results and Discussion

### Temperature-Responsive Behavior of the Branched ELPs

**Figure 1:**
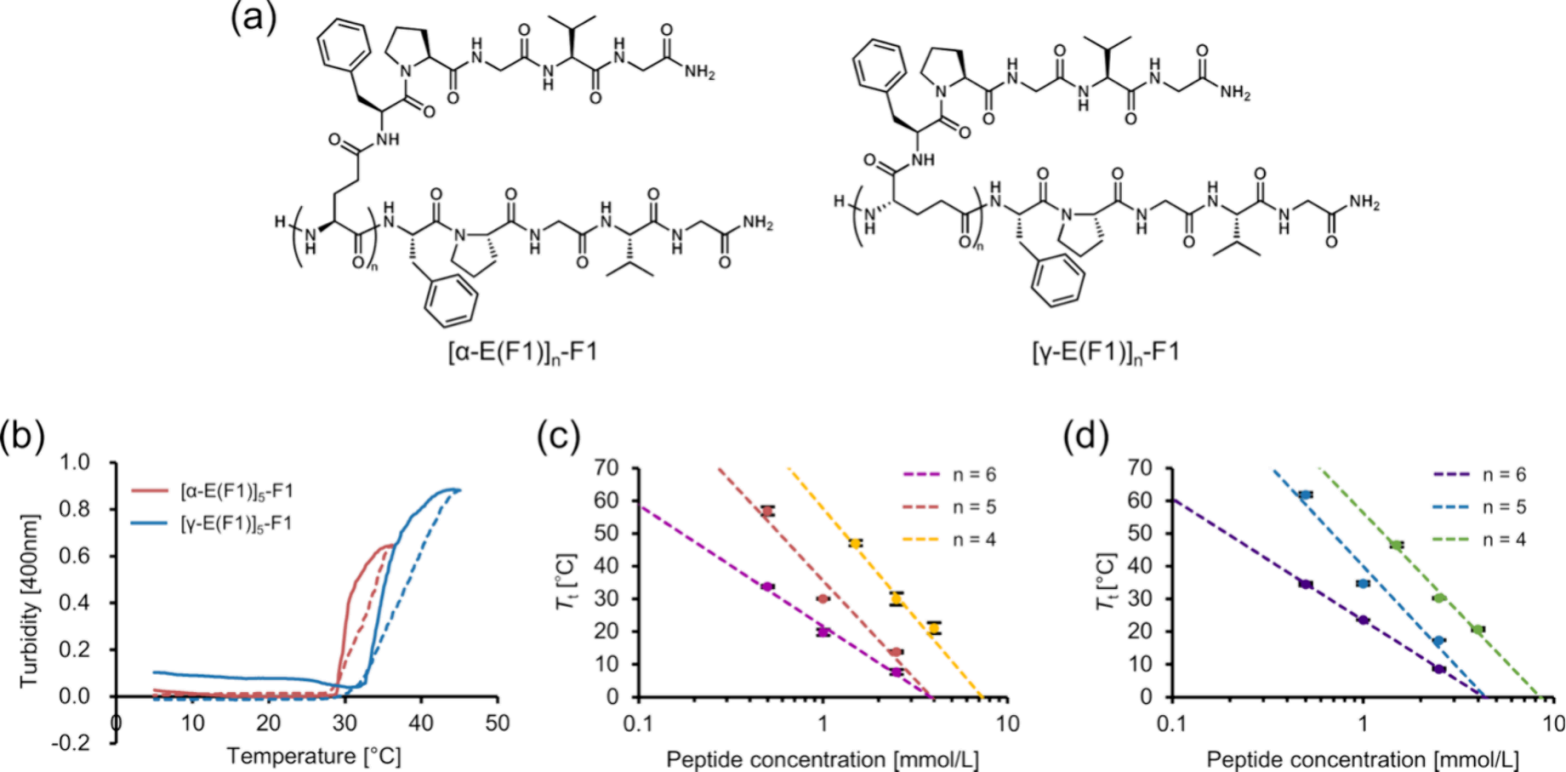
Chemical structures and turbidity measurements of branched ELPs. (a) Chemical structures of [α-E(F1)]*_n_*-F1 (left) and [γ-E(F1)]*_n_*-F1 (right), (b) turbidity measurements of the branched ELPs (*n* = 5) at 1 mM, (c) *T*_t_ vs concentration relationship of [α-E(F1)]_*n*_-F1, and (d) *T*_t_ vs concentration relationship of [γ-E(F1)]_*n*_-F1. In (b), the solid and dashed lines represent the turbidity profiles upon heating and cooling, respectively.

Branched ELPs with brush shape were synthesized via a grafting-onto approach^35^ using Fmoc-(E(OH))_*n*_-F1 (*n* = 4–6) as the backbone oligopeptide and F1 as the side component. The final deprotection of Fmoc-[E(F1)]_*n*_-F1 yielded the target N-terminal free branched ELPs, [α-E(F1)]_*n*_-F1 and [γ-E(F1)]_*n*_-F1 (Figure 1a). The purity of each molecule is shown in Figure S1, and the yield, retention time, and *m*/*z* values are summarized in Table S1. Higher yields were observed for [α-E(F1)]*_n_*-F1 than for [γ-E(F1)]_*n*_-F1. This result may be because oligo-α-Glu provides carboxyl groups that are more distanced from the backbone chains, which facilitate conjugation with F1 chains.

First, the LCST-like behavior of the branched ELPs was investigated by using turbidity measurements. As expected, branched ELPs comprising F1 chains exhibited LCST-like behavior in phosphate buffer, where 1 M NaCl, a salt and salt concentration commonly used in the inverse transition cycling,^9^ was included to facilitate the phase transitions of ELPs (Figures 1b and S2). At a peptide concentration of 1 mM, [α-E(F1)]_5_-F1 and [γ-E(F1)]_5_-F1 showed LCST-like behavior at approximately 30 and 35 °C, respectively (Figure 1b). The obtained *T*_t_ values at different peptide concentrations are shown in Figure 1c,d and Table 1. No significant differences were observed between the *T*_t_ values of [α-E(F1)]*_n_*-F1 and [γ-E(F1)]*_n_*-F1 having the same *n* value (the same number of F1 chains). The concentration dependency of *T*_t_ values showed that the *T*_t_ values of each branched ELPs fit the equation *T*_t_ = *a* ln(*C*) + *b*,^19^ which was originally demonstrated to describe the relationship between *T*_t_ values and concentrations of linear ELPs (Figure 1c,d). A higher concentration and increasing number of F1 chains resulted in lower *T*_t_ values and reduced peptide concentrations required for LCST-like behavior, respectively. These *T*_t_–concentration relationships will help determine which peptide to use and at what concentration to prepare for LCST-like behavior with a target *T*_t_ value. Collectively, these results demonstrate the similarity between the LCST-like behavior of these branched ELPs and those of conventional linear ELPs.

**Table 1:** *T*_t_ Values of the Branched ELPs for the LCST-like Behavior

	*n*	concentration (mM)	*T*_t_ (°C)^a^
[α-E(F1)]_*n*_-F1	4	4	21.12 ± 1.68
		2.5	29.98 ± 1.82
		1.5	47.04 ± 0.81
	5	2.5	13.86 ± 0.33
		1	30.11 ± 0.15
		0.5	56.92 ± 1.28
	6	2.5	7.68 ± 0.80
		1	19.76 ± 0.97
		0.5	33.80 ± 0.38
[γ-E(F1)]_*n*_-F1	4	4	20.75 ± 0.47
		2.5	30.25 ± 0.23
		1.5	46.52 ± 0.67
	5	2.5	17.38 ± 0.10
		1	34.73 ± 0.52
		0.5	61.87 ± 0.60
	6	2.5	8.63 ± 0.31
		1	23.66 ± 0.06
		0.5	34.56 ± 0.48

a *T*_t_ values are shown with standard error.

**Figure 2:**
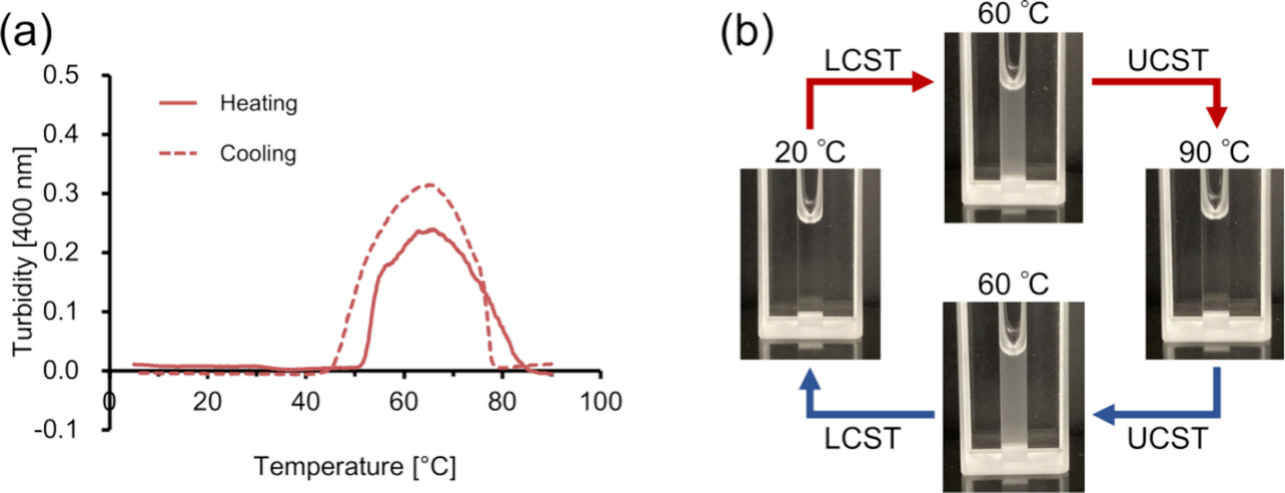
LCST-like and UCST-like behavior of branched ELPs at 0.5 mM. (a) Turbidity measurement of [α-E(F1)]_5_-F1 at 0.5 mM and (b) images of [α-E(F1)]_5_-F1 at 0.5 mM upon temperature changes.

In addition to the LCST-like behavior, branched ELPs with *n* = 5 at 0.5 mM showed solubility at high temperatures after the LCST-type transition (Figures 2 and S3). When cooling them from 90 °C, we observed a turbidity increase. These results show that [α-E(F1)]_5_-F1 and [γ-E(F1)]_5_-F1 at 0.5 mM can exhibit both LCST- and UCST-like behavior. At peptide concentrations higher than 0.5 mM, we observed aggregates at 90 °C, which precipitated at the bottom of a measurement cell and were not soluble even after mixing (Figure S3). This result suggests that peptide concentration is an important factor that controls peptide solubility and insolubility for UCST-like behavior. With regard to brush polymers bearing elastin-based side chains, poly(phenylacetylene) with VPGVG chains has been reported to exhibit UCST-like behavior.^36^ Because polymers with UCST-like behavior in aqueous systems have been relatively less frequently identified,^37−39^ these branched ELPs may contribute to the development and application of molecules with UCST-like behavior. Molecules exhibiting both LCST- and UCST-like behaviors can offer options with distinct temperature ranges for solubilizing the target compounds in a given solution. Moreover, when the temperature is set to above LCST or below UCST, target compounds may become insoluble and can be purified. Taken together, our results highlight the advantages of developing branched ELPs for their unique temperature-responsiveness.

### Morphological Properties of the Branched ELPs

ELPs form coacervates through LCST-like behavior. To date, various architectures such as spherical, cylindrical, or worm-like micelles, as well as nanofibers, have been obtained through the modulation of amino acid composition, pH, or salt conditions and conjugation with other molecules.^40−43^ Optical microscopy and DLS were used to gain insight into the coacervates of the branched short ELPs.

**Figure 3:**
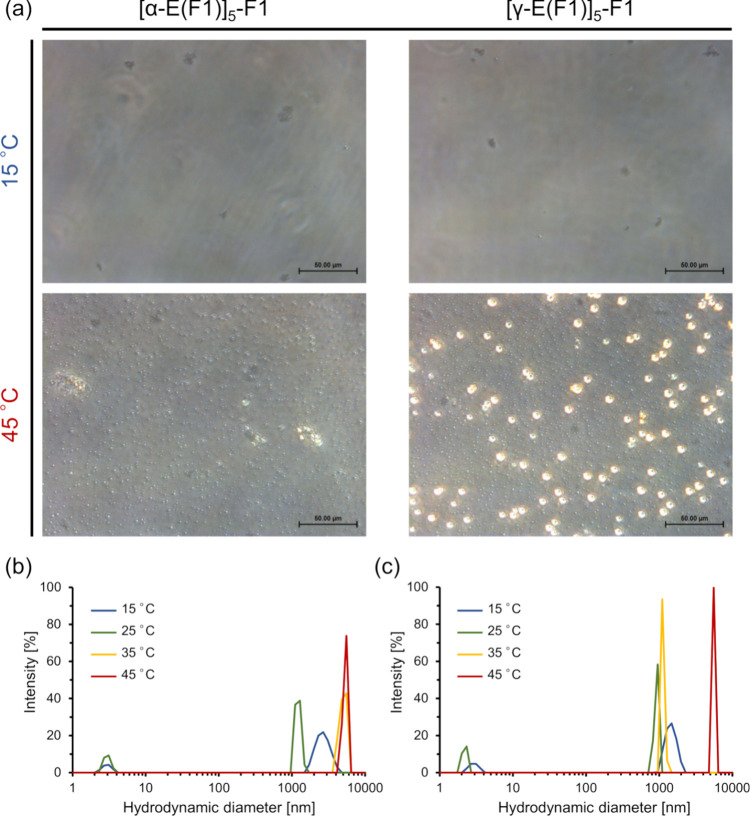
Morphology characterization of branched ELPs. (a) Microscopy images of [α-E(F1)]_5_-F1 and [γ-E(F1)]_5_-F1 taken 5 min after equilibration, (b) DLS results of [α-E(F1)]_5_-F1 at 1 mM, and (c) DLS results of [γ-E(F1)]_5_-F1 at 1 mM. Scale bars indicate 50 μm.

First, samples were prepared at conditions wherein the peptides show LCST-like behavior with *T*_t_ around 30 °C (*n* = 4, 2.5 mM; *n* = 5, 1 mM; *n* = 6, 0.5 mM). For all the peptides, few coacervates were observed by optical microscopy at 15 °C (below *T*_t_), whereas spherical coacervates were observed at 45 °C (above *T*_t_) (Figures 3a and S4–S9). When the temperature returned to 15 °C, the coacervates disappeared. The DLS results showed that the hydrodynamic diameter increased when the temperature reached approximately *T*_t_ (around 30 °C) for the LCST-like behavior (Figures 3b,c and S10). At 15–25 °C, the hydrodynamic diameters ranged from several nanometers to several micrometers. Given the *R*_g_ values of 0.88–0.96 nm obtained from the MD simulations shown below, the hydrodynamic diameters of a single monomeric molecule would be expected to fall within the nanometer range. Therefore, the peaks observed at several nanometers likely correspond to the sizes of the monomers. When the temperature was raised to approximately or above *T*_t_, the hydrodynamic diameters were predominantly several micrometers in size. These results suggest that the ELPs are hydrated but exist as both monomers and multimers below *T*_t_, and these monomers and preformed multimers further self-assemble to form larger particles above *T*_t_. These stepwise particle formations have also been previously reported for linear ELPs,^44^ confirming another similarity in the LCST-like behavior between branched and linear ELPs.

**Figure 4:**
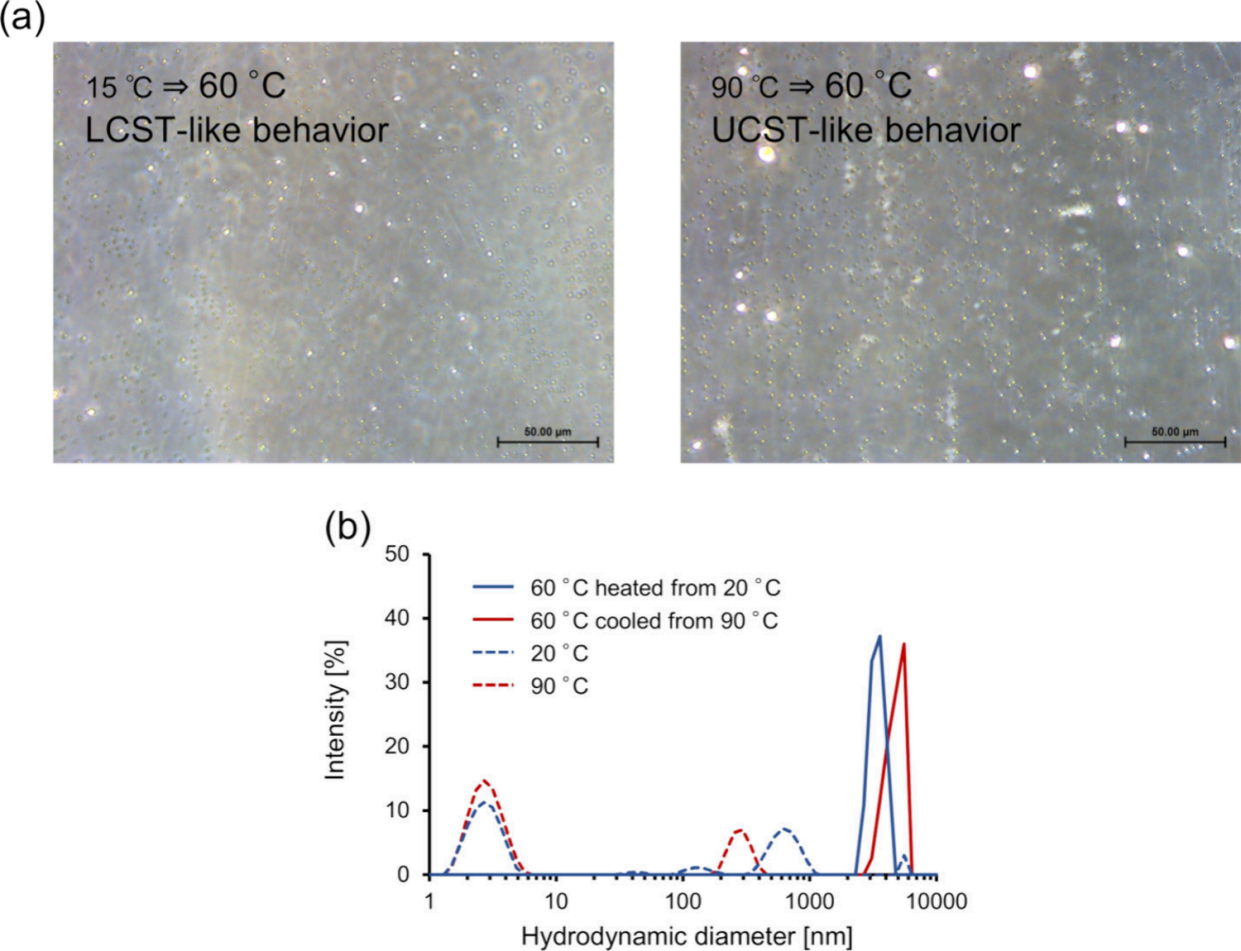
Morphology characterization of [α-E(F1)]_5_-F1 at a concentration of 0.5 mM. (a) Microscopy images of [α-E(F1)]_5_-F1 at 0.5 mM at 60 °C heated from 15 °C (left) and cooled from 90 °C (right) taken 5 min after equilibration and (b) DLS results of [α-E(F1)]_5_-F1 at 0.5 mM. Scale bars indicate 50 μm.

Additionally, aggregates formed through LCST- and UCST-type transitions were also observed for [α-E(F1)]_5_-F1 at 0.5 mM (Figures 4a and S12). DLS results showed larger hydrodynamic diameters at 60 °C compared to the ones at 20 or 90 °C, which confirms that larger particles are formed within a specific temperature range through LCST and UCST transitions (Figures 4b and S13).

### Structural Changes Associated with Temperature Changes

For decades, the structural properties of ELPs in response to temperature changes have been explored using experimental and computational methods.^45^ Nonetheless, conflicting findings have arisen, even for ELPs with a (VPGVG)_*n*_ sequence. Some studies have demonstrated higher preferences for β-turn formation above the LCST,^46−50^ emphasizing the importance of Pro-Gly sequence for the self-assembly of ELPs. However, others have revealed highly disordered structures both below and above the LCST.^51−54^ Recent studies have considered ELPs as intrinsically disordered proteins, which exhibit their functionality without forming a single orderly structure.^55,56^ With regard to the ELPs with a (FPGVG)_*n*_ sequence, higher contents of β-sheets and β-turns have been observed around and above LCST. However, these findings have mostly been obtained for linear Fn molecules,^57,58^ and the structural characteristics of branched molecules are not clear. Therefore, to gain insight into the structural aspects of the branched ELPs ([α-E(F1)]_5_-F1 and [γ-E(F1)]_5_-F1), the structural changes were investigated by CD measurements, ThT assay, and MD simulation.

**Figure 5:**
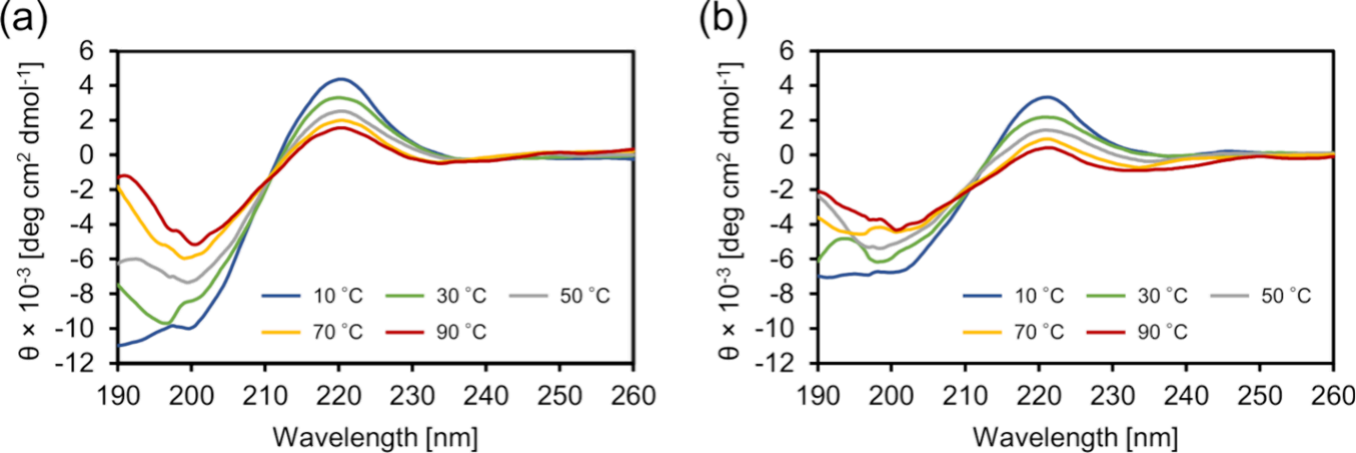
CD spectra of branched ELPs. (a) CD spectra of [α-E(F1)]_5_-F1 upon heating and (b) CD spectra of [γ-E(F1)]_5_-F1 upon heating.

The obtained CD spectra were considered to primarily reflect the structural changes associated with LCST-like behavior, considering that a lower concentration and ionic strength lead to higher *T*_t_ values for LCST-like behavior (possibly resulting in the absence of coacervation behavior). At lower temperatures, the CD spectra of the branched ELPs showed a negative peak at 200 nm and a positive peak at 220 nm, which were assigned to the PPII-like helical structure (Figure 5). These spectral patterns have been reported to distinguish the structure from disordered structures, which typically exhibit small negative peaks at approximately 220 nm and large negative peaks at approximately 195 nm.^59^ As the temperature increased, the intensity of these peaks decreased, and a small shoulder peak emerged at 205 nm, which indicates the formation of β-sheet and β-turn structures. Additionally, an isodichroic point at 208 nm was observed, indicating a structural transition. These CD spectral patterns suggested an increase in β-structures with the temperature increase. Additionally, the reversibility of the secondary structures was confirmed by the CD spectra of the cooling process (Figure S15). Previous studies also observed these structural patterns in ELP analogues with oligomeric F*n* chains.^24,27^ The spectral intensity of [α-E(F1)]_5_-F1 at a specific temperature was larger than that of [γ-E(F1)]_5_-F1. This disparity indicates that the former is a more rigid molecule than the latter.^60^ These properties could be expected from the difference in the flexibility of the backbone structure and were also reflected in the alteration in the *R*_g_ values with temperature changes in the MD simulation (see below).

**Figure 6:**
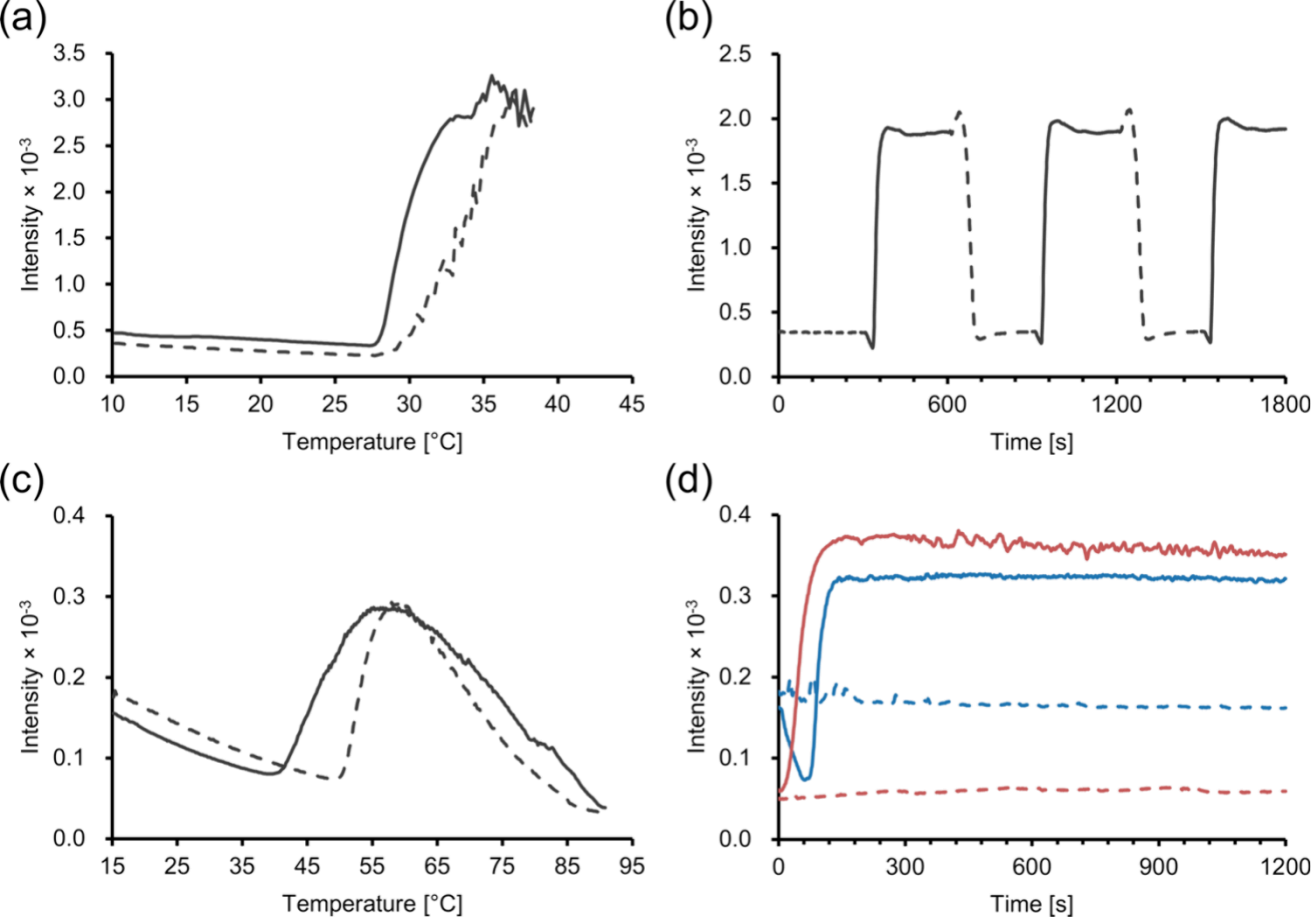
ThT assay of branched ELPs. (a) Temperature-dependent ThT assay of [α-E(F1)]_5_-F1 at 1 mM, (b) time-dependent ThT assay of [α-E(F1)]_5_-F1 at 1 mM with switching temperature, (c) temperature-dependent ThT assay of [α-E(F1)]_5_-F1 at 0.5 mM, and (d) time-dependent ThT assay of [α-E(F1)]_5_-F1 at 0.5 mM. In (a) and (c), the solid and dashed lines represent the fluorescence intensity profiles upon heating and cooling, respectively. In (b), the solid and dashed lines represent fluorescence intensity profiles at 45 and 15 °C, respectively, their onsets representing when the temperature was set at 45 or 15 °C. In (d), the solid blue line represents the fluorescence intensity profile at 60 °C heated from 15 °C, the dashed blue line at 15 °C, the solid red at 60 °C cooled from 90 °C, and the dashed red at 90 °C.

The ThT assay on [α-E(F1)]_5_-F1 at 1 mM showed a sharp increase in fluorescence intensity around *T*_t_ upon heating, indicating an augmentation in β-sheet structure and the formation of amyloid-like structures during LCST-like behavior (Figure 6a).^61^ Upon setting the temperature to above or below the *T*_t_, fluorescence intensity immediately increased or decreased, respectively, demonstrating the reversible formation of the β-sheet structure (Figure 6b). In terms of β-sheet formation for LCST-like behavior, these results aligned with the result obtained from the CD analysis above and findings from previous studies including not only branched F*n* molecules^24,26,27^ but also linear F*n* molecules.^57^ At temperatures below the *T*_t_ for the LCST-like behavior, the fluorescence intensity slightly decreased upon heating (Figure 6a,b), suggesting a decrease in the β-sheet structures.

At 0.5 mM, where the branched peptides exhibited both LCST- and UCST-like behavior, upon heating to 90 °C, the ThT fluorescence intensity increased around the *T*_t_ for LCST-like behavior, reached a maximum around 60 °C, and decreased upon further heating (Figure 6c,d), which corresponds to the aggregation and disaggregation observed in turbidity measurements. Similar to the results at 1 mM, an intensity decrease was also observed at 0.5 mM before LCST-type aggregation (Figure 6c,d). Upon cooling from 90 °C, the intensity increased and reached a maximum at approximately 60 °C and decreased around the *T*_t_ for LCST-like behavior. These results suggest that both the LCST- and UCST-like behaviors of the branched ELPs involve the formation and denaturation of β-sheet structures for aggregation and disaggregation, respectively. The LCST behavior is generally regarded as an entropy-driven process, wherein, for ELPs, the water molecules surrounding the hydrophobic residues of the peptides become less ordered, which is a favorable entropy change above the LCST.^15^ In contrast, UCST behavior is considered to be an enthalpy-driven process, where hydrogen bonds or electrostatic interactions play a key role in the aggregation and disaggregation behavior.^37,38^ While the LCST- and UCST-like behavior of the branched ELP in this study might be entropy- and enthalpy-driven processes, respectively, the ThT assay suggests that both processes entail the increase and decrease of β-sheet structures.

**Figure 7:**
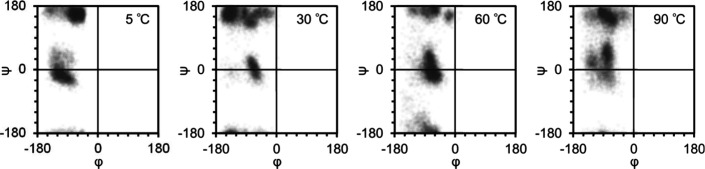
Ramachandran plots of backbone Glu residues of [α-E(F1)]_5_-F1.

To investigate the structural changes of the backbone oligo(Glu), Ramachandran plots were obtained from MD simulations at different temperatures by collecting the dihedral angles of the backbone Glu residues of [α-E(F1)]_5_-F1 (Figure 7). The plot at 5 °C showed a highly populated region around (φ, ψ) = (−60, +150) and a moderately populated region centered around (−100, −20), indicating PPII and helix structures, respectively.^62^ At 30 °C, these structures decreased and regions around (−150, +175) became populated, indicating the presence of β-sheet structures. Subsequently, a decrease in β-sheet structures and an increase in helix structures were observed at 60 °C, which in turn indicates that the increase in ThT fluorescence intensity can be attributed to structural changes in the F1 chains. Upon further heating to 90 °C, helical components decreased, and PPII structures increased again. These structural changes might be attributed to the UCST-like behavior in combination with structural changes in the F1 chains.

### Computational Studies on the Mechanism Underlying Temperature-Responsive Behavior

**Figure 8:**
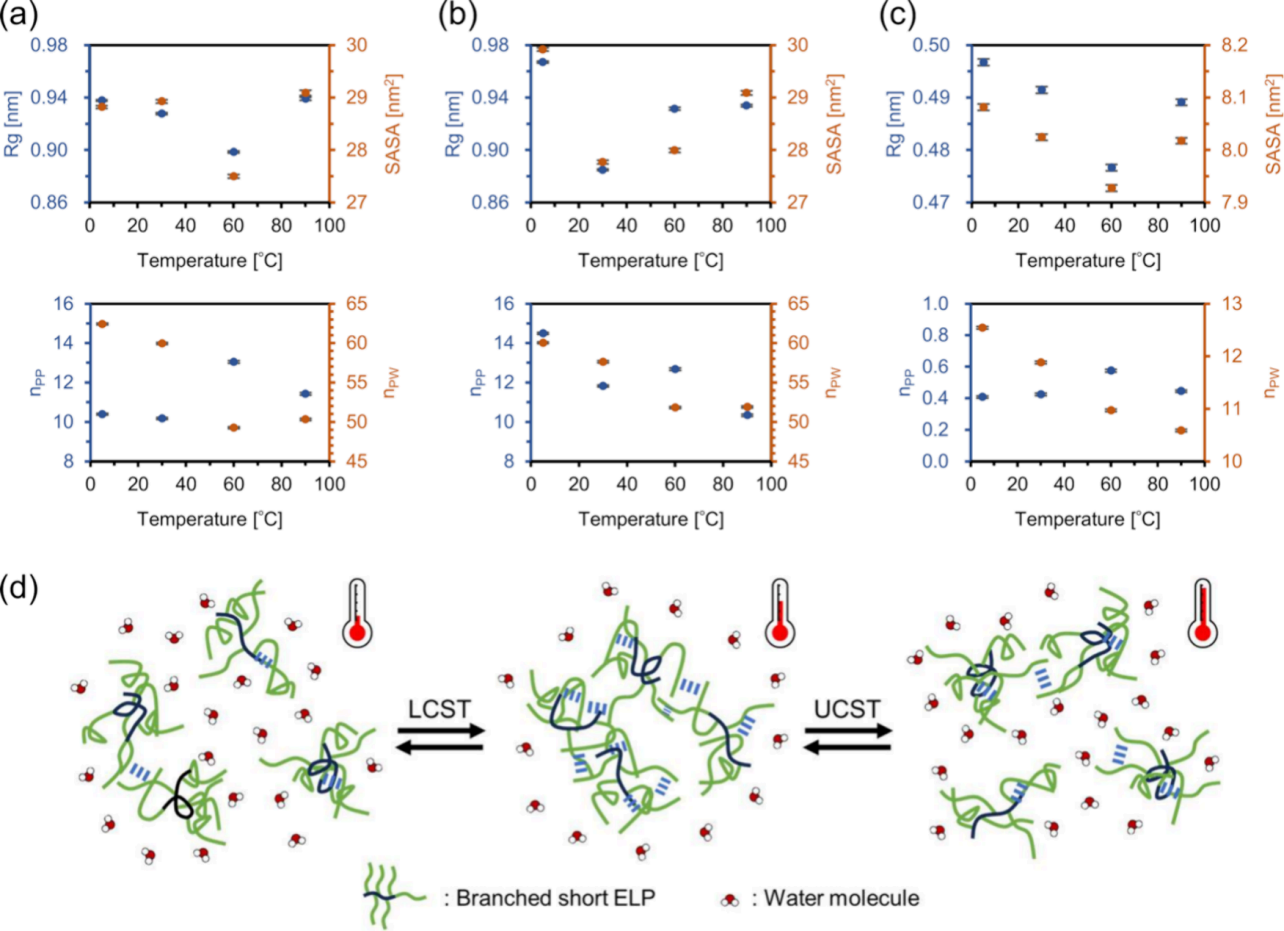
Parameters obtained from MD simulations. (a) [α-E(F1)]_5_-F1, (b) [γ-E(F1)]_5_-F1, and (c) AcF1 and (d) schematic illustration of LCST- and UCST-like behavior. In (a), (b), and (c), the definitions of the parameters are as follows: *R*_g_, radius of gyration; SASA, solvent accessible surface areas; *n*_PP_, number of intramolecular hydrogen bonds; *n*_PW_, number of hydrogen bonds between water and peptide.

To gain further insight into the mechanisms underlying temperature-responsive behavior, the conformation of the peptide and its interactions both within the peptide itself and in the solvent were investigated for three peptides: [α-E(F1)]_5_-F1, [γ-E(F1)]_5_-F1, and Ac-F1-NH_2_ (AcF1). AcF1 represents the side component of the branched ELPs. From the MD simulations performed for a single peptide molecule, *R*_g_, SASA, *n*_PP_, and *n*_PW_ were obtained (Figure 8a–c).

For [α-E(F1)]_5_-F1 and [γ-E(F1)]_5_-F1 (Figure 8a,b), *R*_g_ decreased upon heating to medium temperatures (60 or 30 °C) and increased upon further temperature elevation. These results suggest that the branched ELPs fold upon heating to certain temperatures, which decreases their solubility and facilitates their self-assembly. Subsequent heating leads to unfolding, which increases their solubility and causes the disaggregation of aggregates. The SASA of these peptides showed a trend similar to that of *R*_g_ with changes in temperature. The *n*_PP_ decreased upon heating from 5 to 30 °C, increased at 60 °C, and then decreased again at 90 °C. The high *n*_PP_ value of [γ-E(F1)]_5_-F1 at 5 °C is attributed to the fact that the kinetic energy of peptide molecules is low, allowing molecules to come closer easily, thus making it easier to maintain stable hydrogen bonds. The common feature among these parameters of the branched ELPs, except for the *n*_PP_ value of [γ-E(F1)]_5_-F1 at 5 °C, is that the extremum values were obtained at medium temperatures (30–60 °C). The fluctuation of *n*_PP_ might positively correlate with the formation of β-structures that affected peptide solubility. Therefore, the *n*_PP_ fluctuations at 5–60 °C could correspond to the aggregation/disaggregation behavior upon heating/cooling (LCST-like behavior), and those at 60–90 °C would reflect the disaggregation/aggregation behavior upon heating/cooling (UCST-like behavior). The *n*_PW_ decreased upon heating to 60 °C; however, it slightly increased at 90 °C. The fluctuations of *n*_PP_ and *n*_PW_ indicate that the branched ELPs expose a greater number of hydrophilic sites for nearby water molecules upon heating from 60 to 90 °C, leading to the hydration of the molecule, which ultimately results in the UCST-like behavior.

Overall, our results show that the conformation and solvation of the branched ELPs fluctuate in the range of 5–90 °C with peak parameter values at medium temperatures and suggest that these fluctuations could account for the unique temperature-responsive behavior of the branched short ELPs (Figure 8d). Based on our findings, we suppose that when expecting dual responsiveness from ELP-based molecules, maintaining their self-assembling property to be moderately weak would be crucial because a strong self-assembling property would result in insolubility at high temperatures. Therefore, under conditions employing long peptide chain lengths, highly hydrophobic amino acid compositions, or high peptide concentrations—factors that typically lead to lower *T*_t_ for LCST-like behavior—ELPs may be less likely to exhibit dual-responsive behavior owing to their enhanced self-assembling propensity.

With regard to AcF1, three of the four parameters also showed maximum or minimum values at medium temperatures: among the four temperature points, *R*_g_ and SASA showed the lowest values and *n*_PP_ the highest at 60 °C (Figure 8c). These results indicate that temperature-induced changes in a single F1 chain are conserved in the F1 chains of branched ELPs and contribute to the fluctuation trends in the parameters of the branched ELPs. Notably, folding at medium temperature (approximately 40–60 °C) and unfolding at high temperature (>60 °C) have also been reported for an octapeptide GVG(VPGVG).^63^ Future studies are required to confirm whether similar dual-temperature-responsive behavior can be observed in other types of ELPs, including branched short-chain ELPs as well as branched long-chain, linear short-chain, and linear long-chain ELPs. This is important because turbidity measurements, a common method to examine temperature-responsive behavior, cannot distinguish between “dissolution” and “precipitation” of aggregates at high temperatures. As a result, similar dual-temperature responsiveness could potentially have been overlooked. Therefore, further studies are required to investigate the mechanism underlying the temperature-responsiveness and determine whether the properties of branched and short-chain ELPs are crucial for dual responsiveness.

## Conclusion

In this study, we investigated the temperature-responsive properties of branched ELPs composed of F1 side components and oligo(Glu) backbone structures. These branched ELPs exhibited LCST-like behavior with concentration dependency, secondary structural features, and morphological properties similar to those of conventional linear ELPs. In addition to the LCST-like behavior, the branched ELPs with six F1 chains showed UCST-like behavior at 0.5 mM, which is rarely observed in ELPs. These temperature-responsive behavior were attributed to the alterations in the peptide conformation and hydration states across temperatures from 5 to 90 °C. Therefore, this study demonstrates the unique temperature-responsiveness of branched short ELPs, shedding new light on the future development and utilization of ELPs with desired properties.

## Supporting Information Available

Detailed procedure for MD simulations, yield, *R*_t_, and *m*/*z* values of the branched ELPs (Table S1), UPLC-MS analysis of the branched ELPs (Figure S1), turbidity measurements of the branched ELPs (Figures S2 and S3), microscopy images (Figures S4–S9 and S12), DLS measurements (Figures S10 and S13), DLS autocorrelation curves (Figures S11 and S14), and CD spectra for cooling process (Figure S15) (PDF)

## Abbreviations

CD: circular dichroism
COMU: (1-cyano-2-ethoxy-2-oxoethylidenaminooxy) dimethylaminomorpholinocarbenium hexafluorophosphate
DIPEA: *N*,*N*-diisopropylethylamine
DLS: dynamic light scattering
DMF: *N*,*N*-dimethylformamide
ELP: elastin-like peptide
ESI: electrospray ionization
Fmoc: 9-fluorenylmethyloxycarbonyl
HBTU: 2-(1*H*-benzotriazole-1-yl)-1,1,3,3-tetramethyluronium hexafluorophosphate
LCST: lower critical solution temperature
MD: molecular dynamics
MS: mass spectrum
*n*_PP_: number of intramolecular hydrogen bonds
*n*_PW_: number of hydrogen bonds between water and peptide
PPII: polyproline type II
*R*_g_: radius of gyration
RP-HPLC: reversed-phase high-performance liquid chromatography
SASA: solvent accessible surface areas
TFA: trifluoroacetic acid
ThT: thioflavin T
TIS: triisopropylsilane
*T*_t_: phase transition temperature
UCST: upper critical solution temperature
UPLC: ultraperformance liquid chromatography.
