# Supplementary material for: Exploring LCST- and UCST-like Behavior of Branched Molecules Bearing Repeat Units of Elastin-like Peptides as Side Components
Source: Biomacromolecules. 2024 Oct 9;25(11):7156–66. doi: 10.1021/acs.biomac.4c00751 (PMC11558673; doi:10.1021/acs.biomac.4c00751)
Supplement: Supplementary file 1 — bm4c00751_si_001.pdf [file bm4c00751_si_001.pdf]

Supplementary information

Exploring LCST- and UCST-like Behavior of Branched Molecules Bearing Repeat Units of Elastin-like Peptides as Side Components

AUTHOR NAMES

Naoki Tanaka,[1] Keitaro Suyama,[2] Keisuke Tomohara,[3] and Takeru Nose[1,2,*]

AUTHOR ADDRESS

[1]Department of Chemistry, Faculty and Graduate School of Science, Kyushu University, Fukuoka 819-0395, Japan.

[2]Faculty of Arts and Science, Kyushu University, Fukuoka 819-0395, Japan.

[3]Faculty and Graduate School of Pharmaceutical Sciences, Kyoto Pharmaceutical University, Kyoto, 607-8412, Japan.

Manuscript Correspondence:
Prof. Takeru Nose
Tel: +81-92-802-6025
Fax: +81-92-802-6025
e-mail: nose@artsci.kyushu-u.ac.jp

Supporting Method

Molecular dynamics (MD) simulation……………………….………………..………………2

Supporting Table and Figures

Table S1. Yield, R_t, and *m/z* values of the branched ELPs………………………………4
Figure S1. UPLC-MS analysis of the branched ELPs……………………………………5
Figure S2. Turbidity measurements of the branched ELPs…………………..…………..6
Figure S3. Turbidity measurements of the branched ELPs………………………………7
Figure S4. Microscopy images of [α-E(F1)]$_4$-F1…………..……………………………8
Figure S5. Microscopy images of [α-E(F1)]$_5$-F1……………..…………………………9
Figure S6. Microscopy images of [α-E(F1)]$_6$-F1…………………………..………...10
Figure S7. Microscopy images of [γ-E(F1)]$_4$-F1…………………………………..…11
Figure S8. Microscopy images of [γ-E(F1)]$_5$-F1…………..……………………………12
Figure S9. Microscopy images of [γ-E(F1)]$_6$-F1…………………...……..……………13
Figure S10. DLS measurements of the branched ELPs for LCST-like behavior.………..14
Figure S11. DLS autocorrelation curves of the branched ELPs for LCST-like behavior...15
Figure S12. Microscopy images of [α-E(F1)]$_5$-F1 at 0.5 mM………………………...16
Figure S13. DLS measurements of [α-E(F1)]$_5$-F1 at 0.5 mM………………...…………17
Figure S14. DLS autocorrelation curves of [α-E(F1)]$_5$-F1 at 0.5 mM……..……………18
Figure S15. CD spectra of the branched ELPs obtained for cooling process…………....19

Abbreviations……….…………………………………………………………...20

Supporting Method

Molecular Dynamics (MD) simulation

MD simulation was carried out by using GROMACS 2019 software with Amber ff99SB-ILDN force field [1]. In this study, the structural dynamics of [α-E(F1)]$_5$-F1 (H-[α-E(FPGVG)]$_5$-FPGVG-NH_2), [γ-E(F1)]$_5$-F1 (H-[γ-E(FPGVG)]$_5$-FPGVG-NH_2), and AcF1 (Ac-FPGVG-NH_2) was analyzed. TIP3P explicit solvent model was used to analyze the interactions between the peptides and water molecules. Initial conformations (.pdb file) were generated by Discovery studio 4.0 software (Dassault Systemes BIOVIA, San Diego, CA, USA) and translated into .gro file by gmx pdb2gmx command in GROMACS. The model of [α-E(F1)]$_5$-F1 and [γ-E(F1)]$_5$-F1 were placed in 7 × 7 × 7 nm^3 cubic box and solvated with explicit TIP3P water molecules by gmx solvate. Consequently, appropriate numbers of water molecules were replaced for 206 of Na^+ and 207 of Cl^- via gmx genion to mimic the experimental condition (1 M NaCl) and neutralize the total charge of the system. In the case of AcF1, the peptide model was placed in a 3 × 3 × 3 nm^3 cubic box, solvated with explicit TIP3P water molecules and then appropriate numbers of water molecules were replaced for 16 of Na^+ and 16 of Cl^-.

The simulation was performed at 278, 303, 333, and 363 K (5, 30, 60, and 90 °C) to consider the change in molecular state due to difference of temperature. These systems contained two minimizations, heating step, equilibrium step, and production step. The first minimization was performed by steepest descent algorithm, using a maximum of 10,000 steps, the maximum step size (emstep) of 0.01 nm, the tolerance (emtol) of 10.0 kJ mol^{-1} nm^{-1}, and no constraints; The second minimization was performed by conjugate gradient algorithm, using a maximum of 20,000 steps, emstep of 0.01 nm, emtol of 10.0 kJ mol^{-1} nm^{-1}, and no constraints; heating step: parameters are 500,000 steps, time step 2 fs, initial temperature 0 K, target temperature (278/303/333/363 K), LINCS constraint to h-bond atoms, annealing type single, annealing npoints 2, and annealing time 0 200; an equilibration step: parameters are 500,000 steps with a time step of 2 fs, target temperature (278/303/333/363 K), LINCS constraint to h-bond atoms, and reference pressure 1.0 bar; and finally, a production step consisting of 15,000,000 steps (30 ns) for [α-E(F1)]$_5$-F1 and [γ-E(F1)]$_5$-F1, and 50,000,000 steps (100 ns) for AcF1, time step 2 fs, target temperature (278/303/333/363 K), pressure coupling decay time (tau-p) = 1.0, LINCS constraint to h-bond atoms, nonbond list radius (rcoulomb, rvdw) = 1.0 nm, nonbond lower cutoff distance (rlist) = 1.0 nm, electrostatics = particle-mesh Ewald (PME) method, dynamics integrator= leapfrog, and random number seed = 1732. Trajectories (30,000 frames for [α-E(F1)]$_5$-F1 and [γ-E(F1)]$_5$-F1, and 100,000 frames for AcF1) were processed by gmx trjcomv command to correct the break in the molecular structure due to periodic boundary

condition. During this process, the number of frames were reduced to 1/10 (3,000 or 10,000 frames). Then, peptide structures were analyzed using the processed trajectory file with omitting the first 10 ns.

Reference

[1] Hess, B.; Kutzner, C.; van der Spoel, D.; Lindahl, E. GROMACS 4: Algorithms for Highly Efficient, Load-Balanced, and Scalable Molecular Simulation. *J. Chem. Theory Comput.* **2008**, *4* (3), 435–447. https://doi.org/10.1021/ct700301q.

Supporting Table and Figures

Table S1. Yield, R_t, and *m/z* values of the branched ELPs.

	n	Yield	R_t[a] (min)	MS (ESI) *m/z*[b] (calcd/found)
[α-E(F1)]$_n$-F1	4	32.07 mg (57%)	2.093	940.08 $[M + 3H]^{3+}$/940.12
	5	39.34 mg (58%)	2.266	1135.30 $[M + 3H]^{3+}$/1135.26
	6	29.56 mg (37%)	2.382	998.14 $[M + 4H]^{4+}$/998.11
[γ-E(F1)]$_n$-F1	4	8.59 mg (15%)	1.860	940.08 $[M + 3H]^{3+}$/940.07
	5	8.72 mg (13%)	2.008	1135.30 $[M + 3H]^{3+}$/1135.25
	6	6.59 mg (8%)	2.127	998.14 $[M + 4H]^{4+}$/998.07

[a] Retention times in UPLC. The solvent system for UPLC consisted of 0.1% formic acid aqueous solution (v/v, solvent A) and 0.1% formic acid in acetonitrile (v/v, solvent B). The peptides were monitored in a linear gradient of solvent B in solvent A (24% to 56%) over 4.23 min.

[b] *m/z* in UPLC-MS. The calculated values are for the average mass.

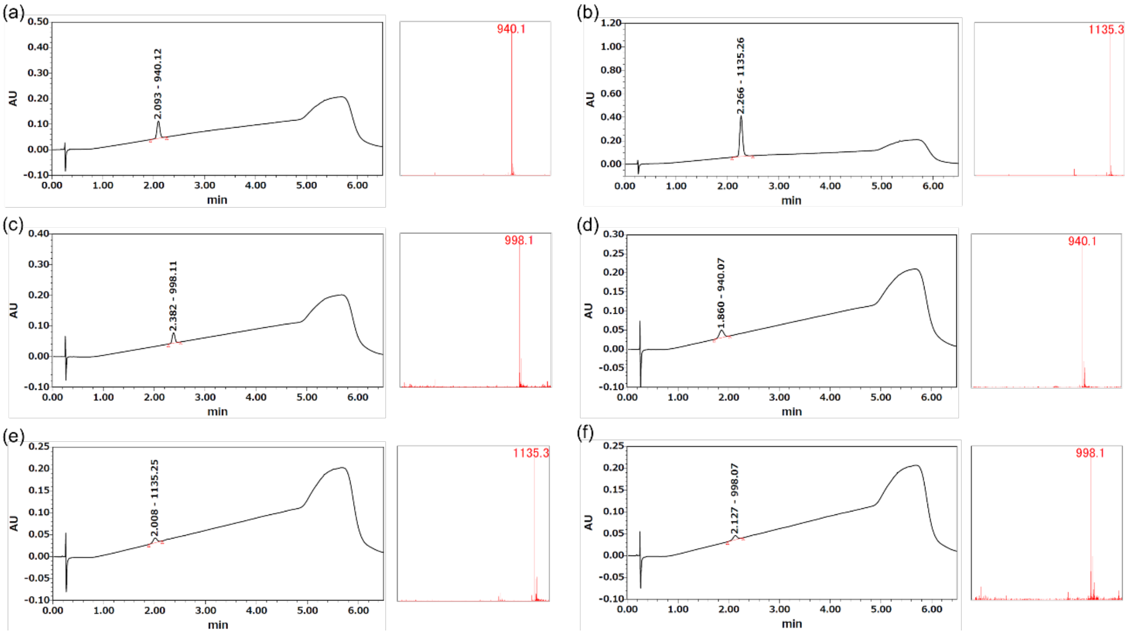

Figure S1. UPLC-MS analysis of the branched ELPs.

(a) [α-E(F1)]$_4$-F1, (b) [α-E(F1)]$_5$-F1, (c) [α-E(F1)]$_6$-F1, (d) [γ-E(F1)]$_4$-F1, (e) [γ-E(F1)]$_5$-F1, and (f) [γ-E(F1)]$_6$-F1. The subscripts for each peak in the left panels indicate the retention time and the *m/z* detected. The right panels show MS spectra of the corresponding peak determined by ACQUITY QDa mass spectrometer. The peptides were monitored in a linear gradient of solvent B in solvent A (24% to 56%) over 4.23 min.

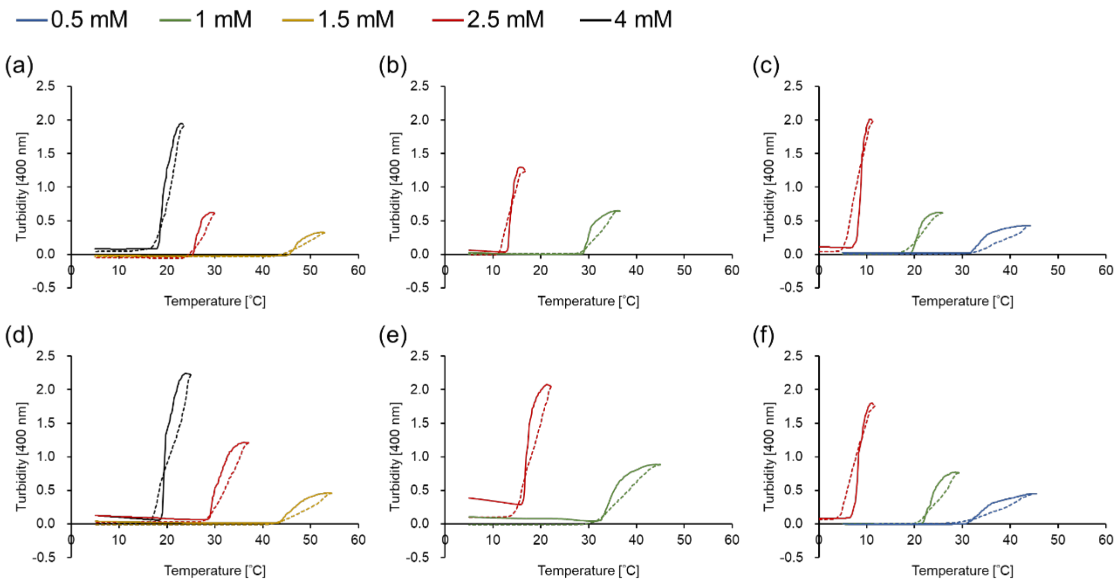

Figure S2. Turbidity measurements of the branched ELPs.

(a) [α-E(F1)]$_4$-F1, (b) [α-E(F1)]$_5$-F1, (c) [α-E(F1)]$_6$-F1, (d) [γ-E(F1)]$_4$-F1, (e) [γ-E(F1)]$_5$-F1, and (f) [γ-E(F1)]$_6$-F1 at various concentrations. For [α-E(F1)]$_5$-F1 and [γ-E(F1)]$_5$-F1 at 0.5 mM, the turbidity measurements are shown in Fig. S3a. Solid and dashed lines represent the turbidity profiles upon heating and cooling, respectively.

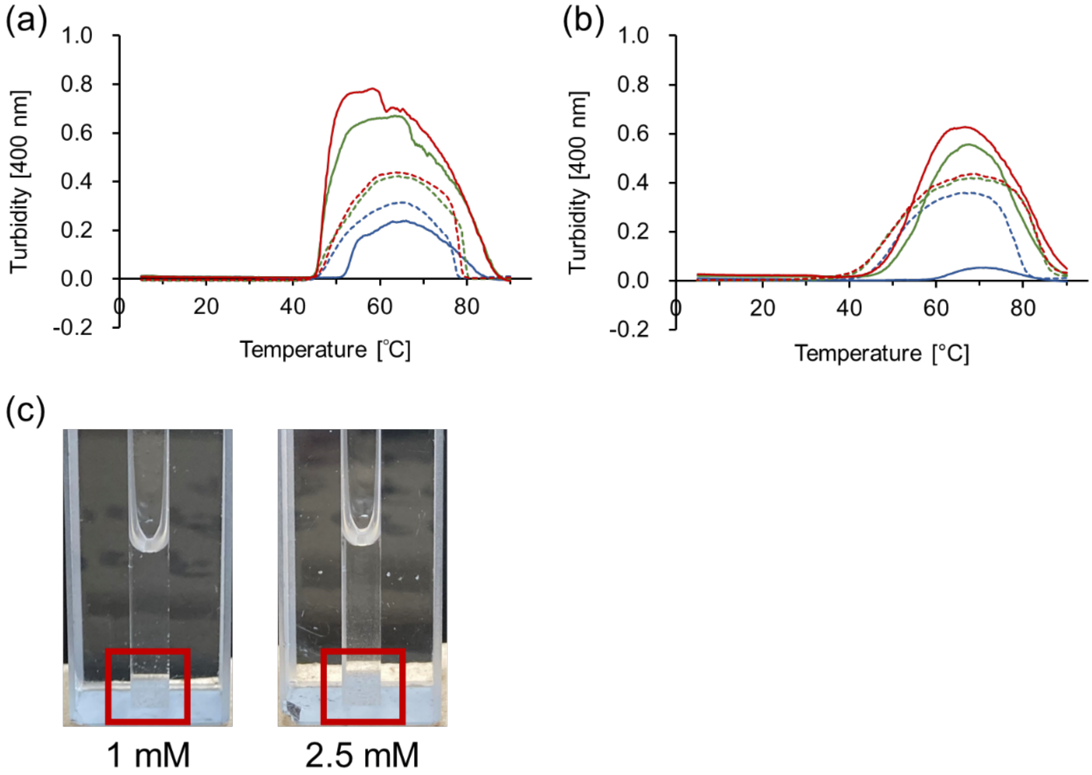

Figure S3. Turbidity measurements of the branched ELPs.

(a) [α-E(F1)]$_5$-F1 at 0.5 mM, (b) [γ-E(F1)]$_5$-F1 at 0.5 mM, and (c) images of [α-E(F1)]$_5$-F1 at 1 mM and 2.5 mM at 90 °C. In (a) and (b), blue, green, and red lines represent the first, second, and third heating-cooling cycle, respectively. Solid and dashed lines represent the turbidity profiles upon heating and cooling, respectively. In (c), insoluble aggregates were observed at the bottom of a measurement cell (highlighted in red frames).

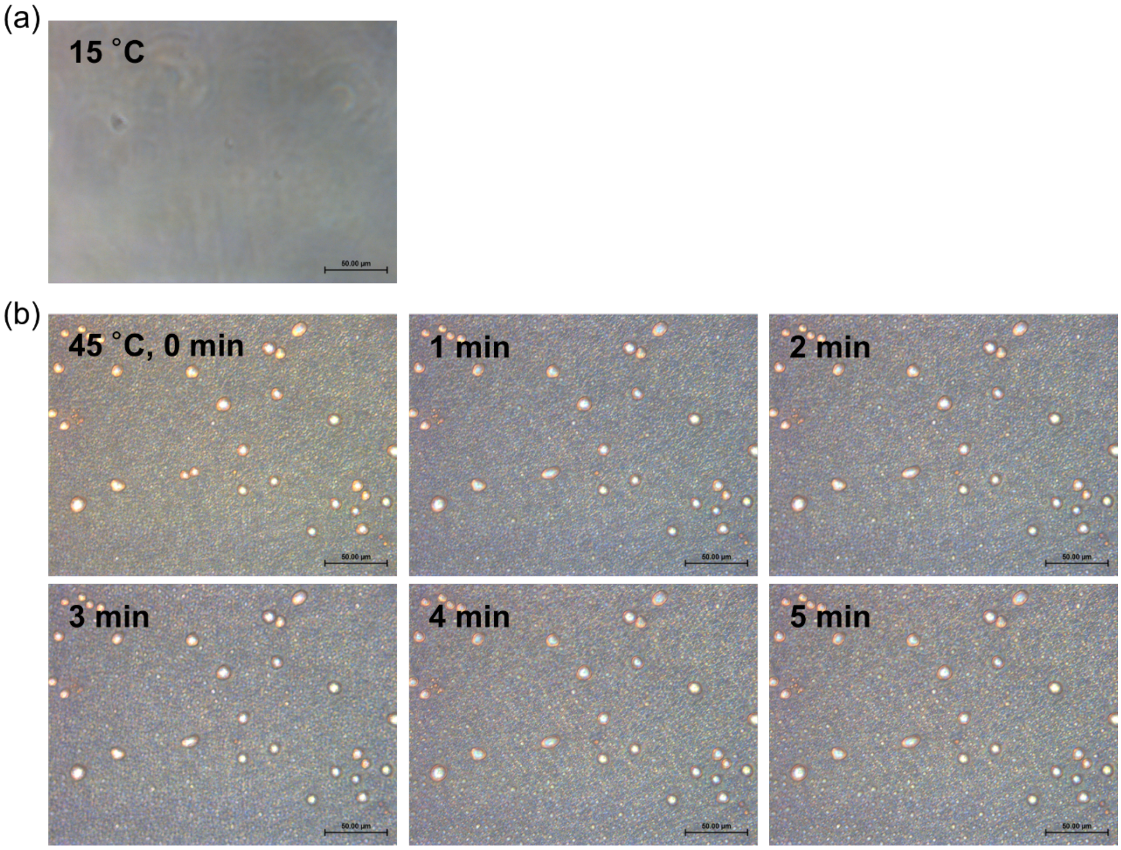

Figure S4. Microscopy images of [α-E(F1)]$_4$-F1.
(a) 15 °C and (b) 45 °C. In (b), time passed after 2 minutes of equilibration is shown. The samples were prepared at 2.5 mM. Scale bars indicate 50 μm.

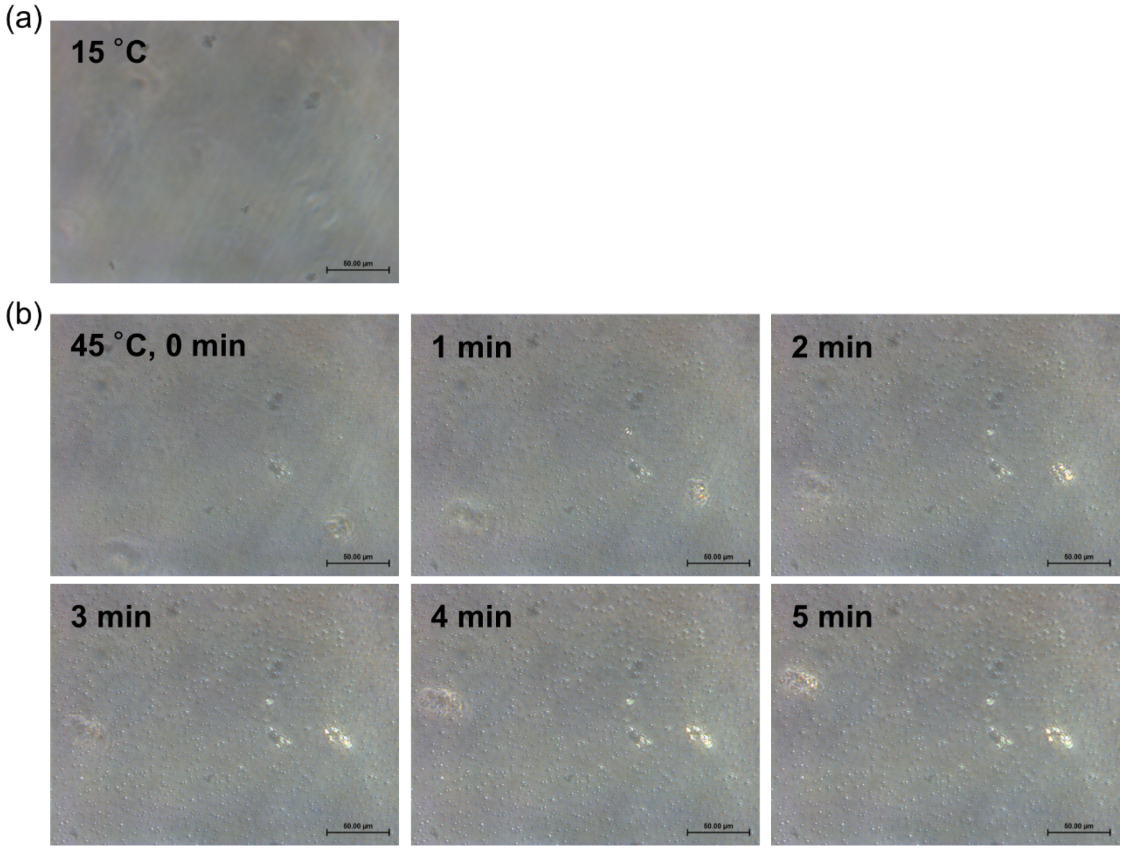

Figure S5. Microscopy images of [α-E(F1)]$_5$-F1.
(a) 15 °C and (b) 45 °C. In (b), time passed after 2 minutes of equilibration is shown. The samples were prepared at 1.0 mM. Scale bars indicate 50 μm.

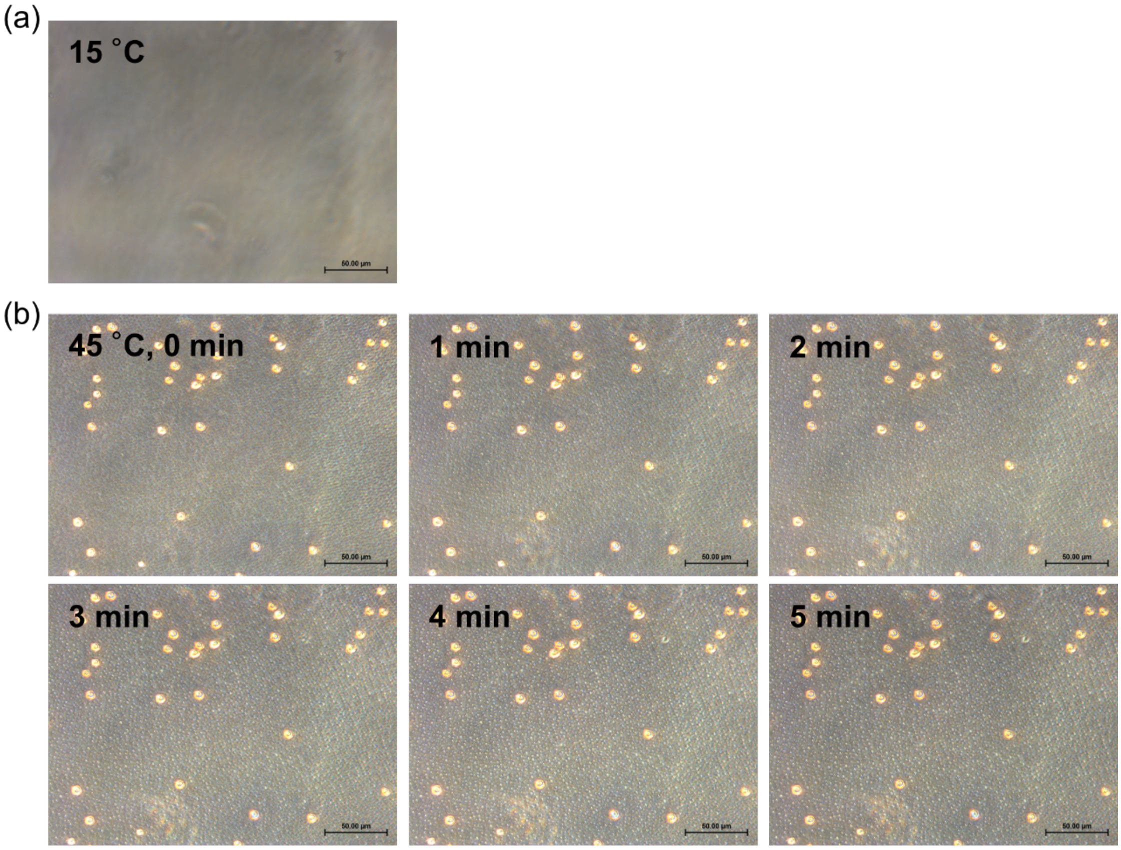

Figure S6. Microscopy images of [α-E(F1)]$_6$-F1.
(a) 15 °C and (b) 45 °C. In (b), time passed after 2 minutes of equilibration is shown. The samples were prepared at 0.5 mM. Scale bars indicate 50 μm.

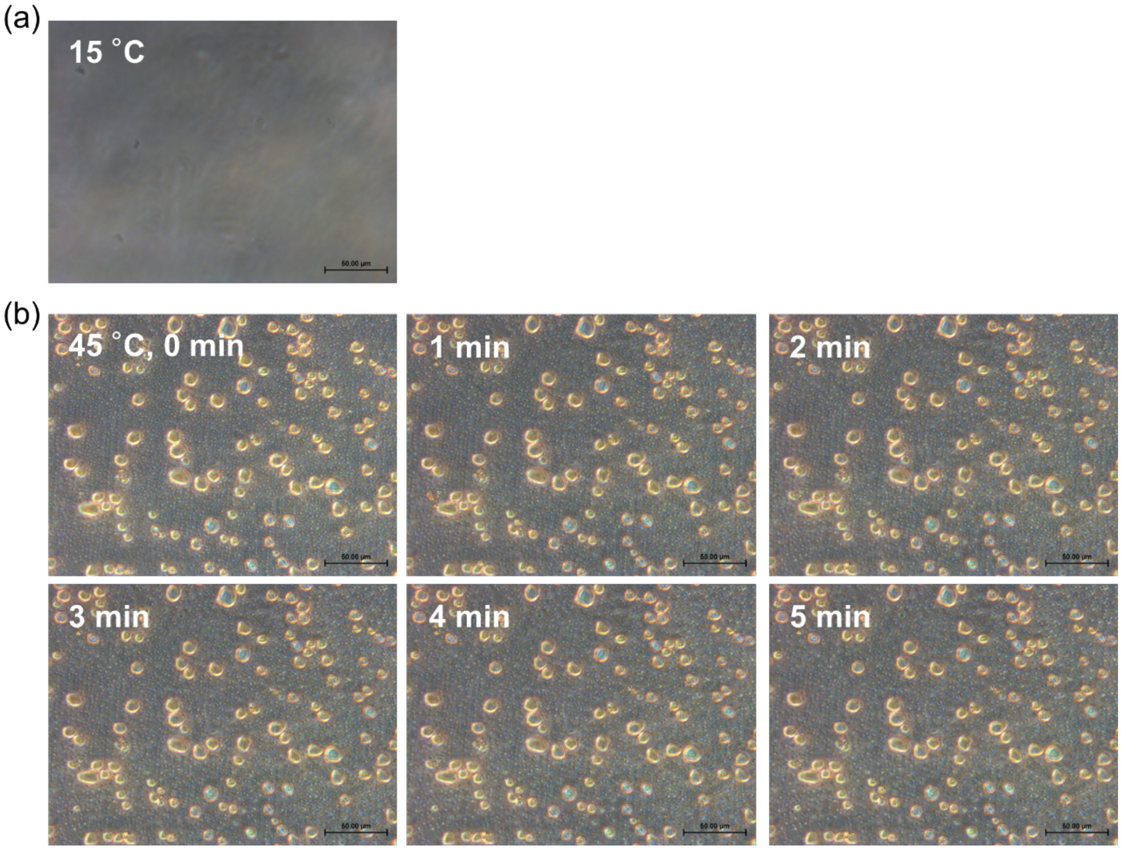

Figure S7. Microscopy images of [γ-E(F1)]$_4$-F1.

(a) 15 °C and (b) 45 °C. In (b), time passed after 2 minutes of equilibration is shown. The samples were prepared at 2.5 mM. Scale bars indicate 50 μm.

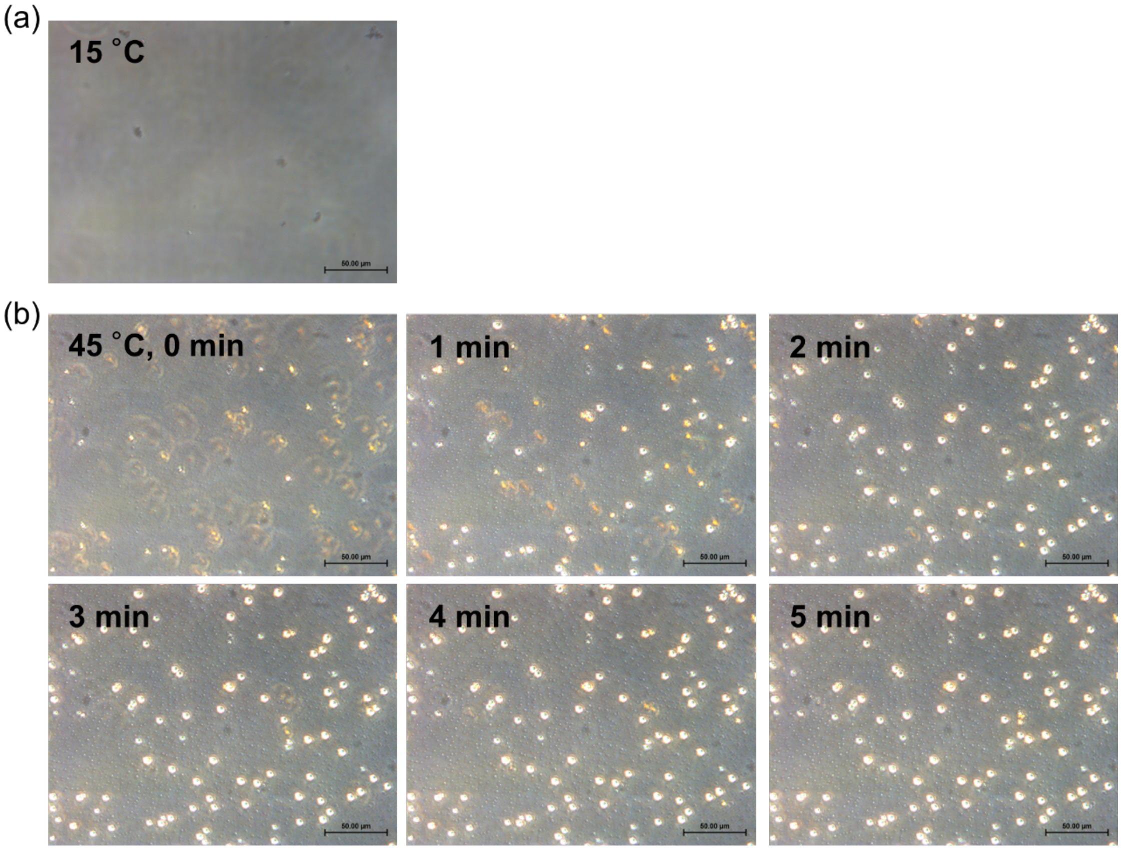

Figure S8. Microscopy images of [γ-E(F1)]$_5$-F1.

(a) 15 °C and (b) 45 °C. In (b), time passed after 2 minutes of equilibration is shown. The samples were prepared at 1.0 mM. Scale bars indicate 50 μm.

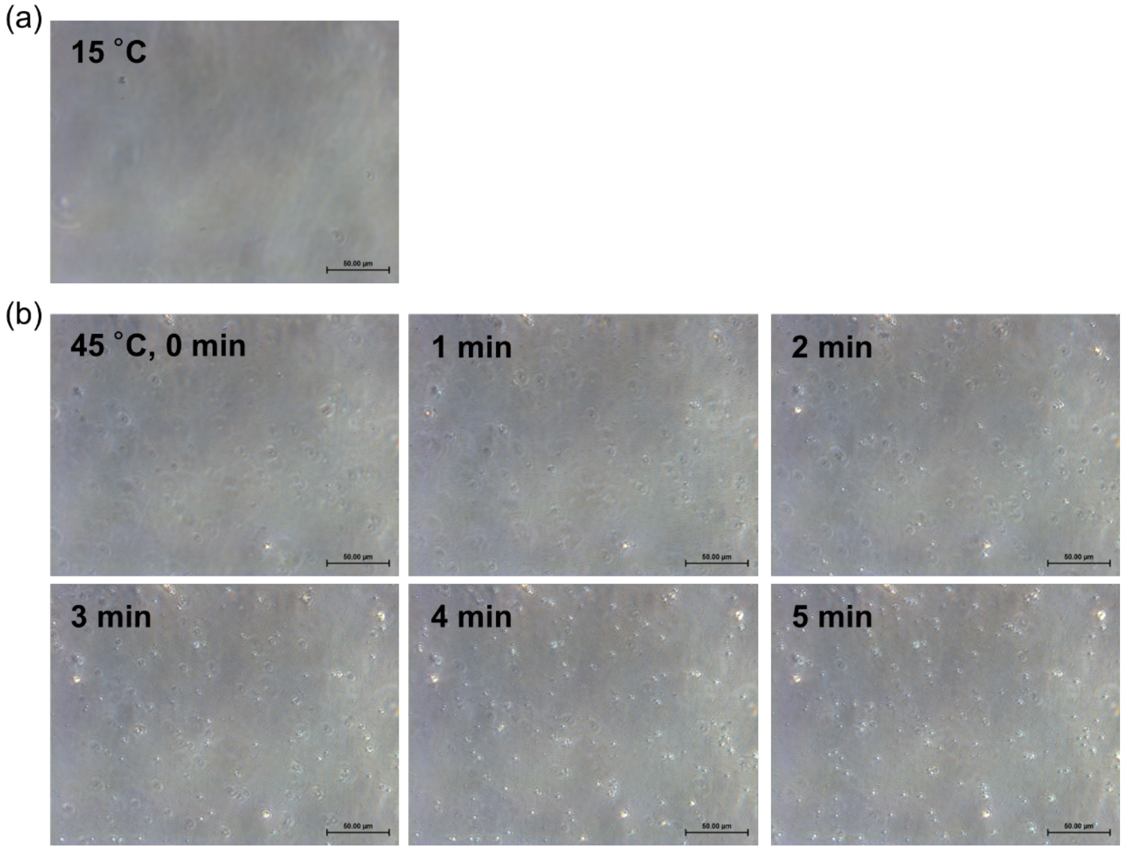

Figure S9. Microscopy images of [γ-E(F1)]$_6$-F1.
(a) 15 °C and (b) 45 °C. In (b), time passed after 2 minutes of equilibration is shown. The samples were prepared at 0.5 mM. Scale bars indicate 50 µm.

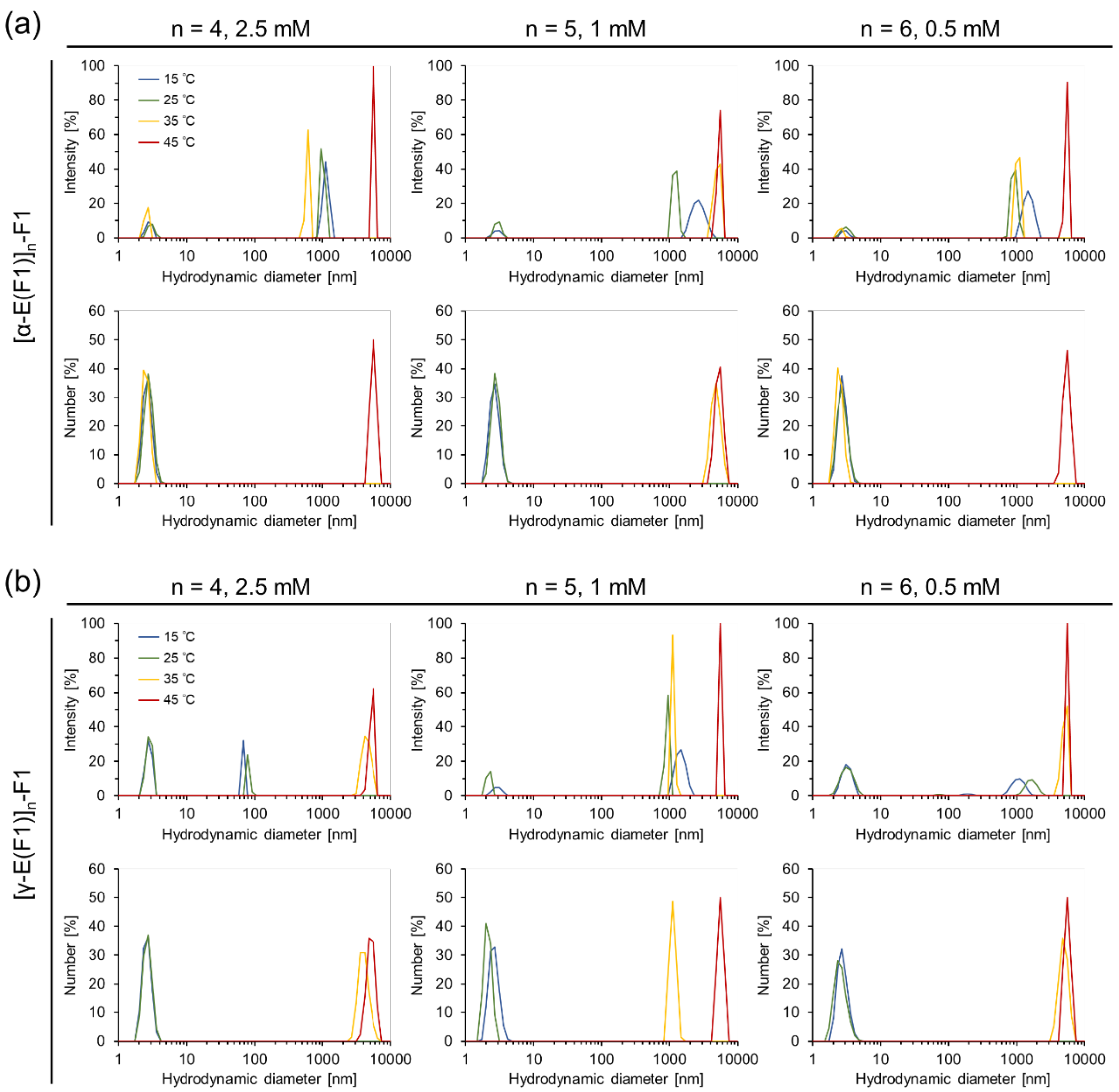

Figure S10. DLS measurements of the branched ELPs for LCST-like behavior.

(a) [α-E(F1)]$_n$-F1 and (b) [γ-E(F1)]$_n$-F1. The results are shown in scattering intensity (upper panels) and particle number (lower panels). The samples were prepared at conditions where the branched ELPs have T_t around 30 °C.

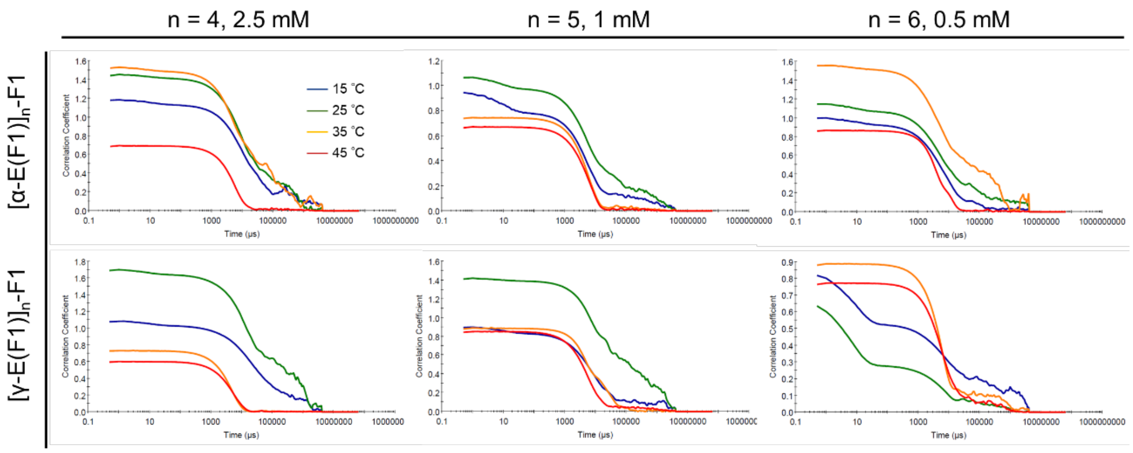

Figure S11. DLS autocorrelation curves of the branched ELPs for LCST-like behavior. The samples were prepared at conditions where the ELPs have T_t around 30 °C.

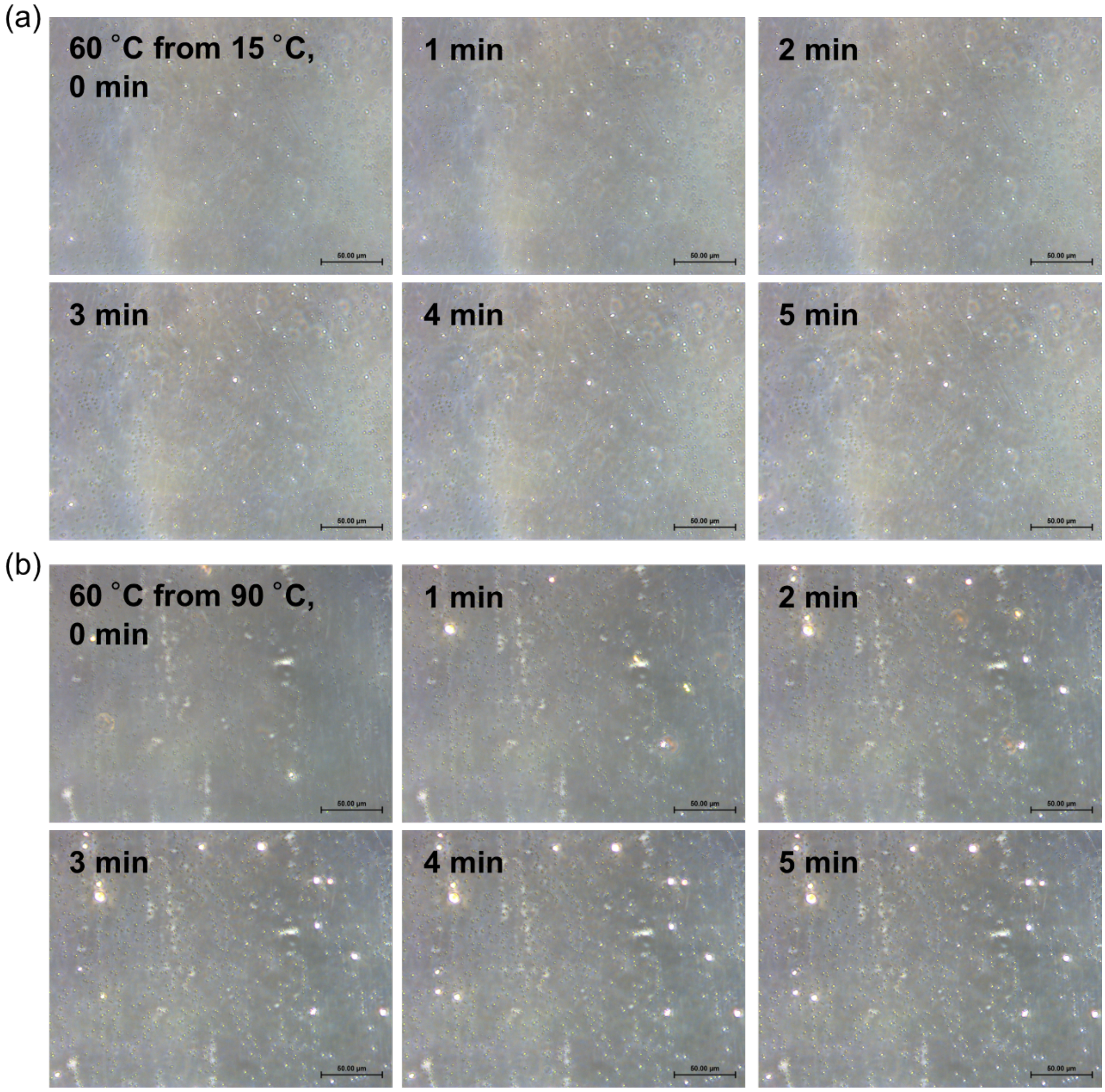

Figure S12. Microscopy images of [α-E(F1)]$_5$-F1 at 0.5 mM.

(a) 60 °C heated from 15 °C (LCST-like behavior) and (b) 60 °C cooled from 90 °C (UCST-like behavior). Time passed after 2 minutes of equilibration is shown. The samples were prepared at 0.5 mM. Scale bars indicate 50 μm.

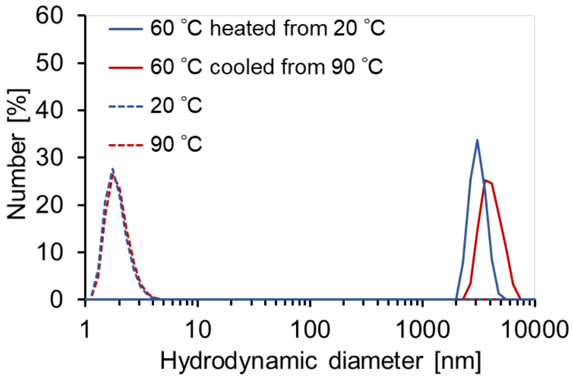

Figure S13. DLS measurements of [α-E(F1)]$_5$-F1 at 0.5 mM.

The results are shown in particle number. The data were converted based on the measurement results of scattered light intensity shown in Figure 4b.

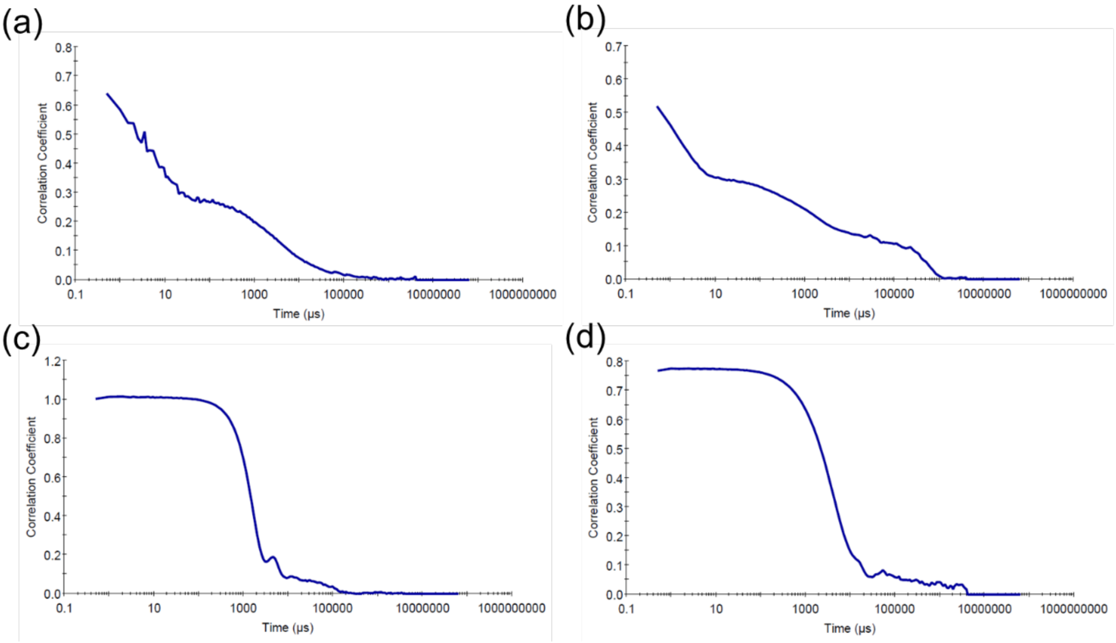

Figure S14. DLS autocorrelation curves of [α-E(F1)]$_5$-F1 at 0.5 mM.
(a) 20 °C, (b) 90 °C, (c) 60 °C heated from 20 °C, and (d) 60 °C cooled from 90 °C.

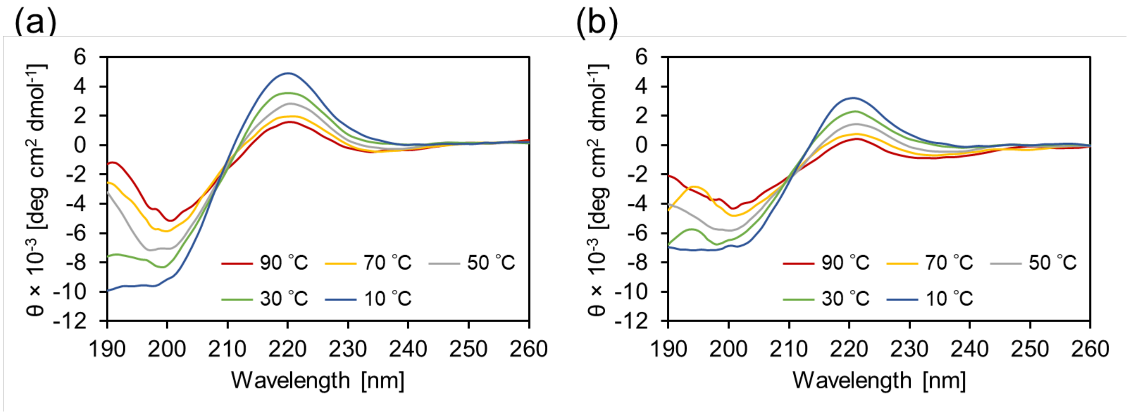

Figure S15. CD spectra of the branched ELPs obtained for cooling process.
(a) [α-E(F1)]$_5$-F1 and (b) [γ-E(F1)]$_5$-F1. Each ELP was prepared at 0.1 mg/mL in filtered phosphate buffer.

Abbreviations

CD, circular dichroism; DLS, dynamic light scattering; ELP, elastin-like peptide; ESI, electrospray ionization; LCST, lower critical solution temperature; MD, molecular dynamics; MS, mass spectrum; R_t, retention time; T_t, phase transition temperature; UCST, upper critical solution temperature; UPLC, ultra-performance liquid chromatography.
